# MALDI-TOF mass spectrometry: an emerging technology for microbial identification and diagnosis

**DOI:** 10.3389/fmicb.2015.00791

**Published:** 2015-08-05

**Authors:** Neelja Singhal, Manish Kumar, Pawan K. Kanaujia, Jugsharan S. Virdi

**Affiliations:** ^1^Department of Microbiology, University of DelhiNew Delhi, India; ^2^Department of Biophysics, University of DelhiNew Delhi, India

**Keywords:** bacterial identification, fungi, MALDI-TOF MS, peptide mass fingerprint, proteomics, viruses

## Abstract

Currently microorganisms are best identified using 16S rRNA and 18S rRNA gene sequencing. However, in recent years matrix assisted laser desorption ionization-time of flight mass spectrometry (MALDI-TOF MS) has emerged as a potential tool for microbial identification and diagnosis. During the MALDI-TOF MS process, microbes are identified using either intact cells or cell extracts. The process is rapid, sensitive, and economical in terms of both labor and costs involved. The technology has been readily imbibed by microbiologists who have reported usage of MALDI-TOF MS for a number of purposes like, microbial identification and strain typing, epidemiological studies, detection of biological warfare agents, detection of water- and food-borne pathogens, detection of antibiotic resistance and detection of blood and urinary tract pathogens etc. The limitation of the technology is that identification of new isolates is possible only if the spectral database contains peptide mass fingerprints of the type strains of specific genera/species/subspecies/strains. This review provides an overview of the status and recent applications of mass spectrometry for microbial identification. It also explores the usefulness of this exciting new technology for diagnosis of diseases caused by bacteria, viruses, and fungi.

## Introduction

The genomic information within a microbial cell translates into more than 2000 proteins, a substantial number of which can be studied using proteomics (Wasinger et al., 1995). It is estimated that for genomes which contain less than 1000 genes, more than 50% of predicted proteome may be identified from the genome. Similarly 30 and 10% predicted proteome may be identified from genomes which carry ca. 2500 and ca. 4000 genes respectively (Jungblut and Hecker, 2004). Thus, a microbial genome containing 600–7000 predicted genes represents a medium-sized complex system where application of proteomics may provide knowledge of a substantial part of the microbe’s proteome.

Characterization and differentiation of microbial proteome developed and progressed with both gel-based and gel-free protein separation methods. SDS-PAGE of whole cell proteins, coupled with computer assisted analysis was used in a few studies for identification and classification of microorganisms (Vandamme et al., 1996). When performed under standardized conditions, this technique was reported to be quite reproducible. The species identification showed an excellent correlation with DNA–DNA hybridization (Vandamme et al., 1996). However, SDS-PAGE protein profiling did not become popular among microbiologists. This might be attributed to: (i) lack of extensive databases for identification of unknown microorganisms, (ii) requirement of highly standardized conditions which included growth of unknown microorganisms on identical media, standardized electrophoretic conditions, staining procedures, and subsequent pattern analysis, (iii) the technique was not precise enough to differentiate highly similar strains. Two-dimensional gel electrophoresis (2-DE) also failed to become popular among microbiologists since it was a laborious, hands-on method, even after ready availability of precast commercially produced gels and improved gel analysis softwares (Cash, 2009). The merits and demerits of other quantitative proteomic approaches have been reviewed elsewhere (Tiwari and Tiwari, 2014).

Analysis of cellular proteome is a method which occupies an intermediary position with respect to the phenotypic–genotypic dichotomy, since the proteins analyzed reflect gene products and metabolic functions. The various methods which have been commonly used for detection of microorganisms in a clinical microbiology set-up, with their respective advantages and disadvantages have been listed in **Table 1**. The increasing use of DNA fingerprinting methods in the last two decades or so has unequivocally established their applicability and utility for identification and classification of microorganisms. However, automation in proteomics technology, in recent years, has increased its throughput and potential use for a number of microbiological purposes like, strain typing and epidemiological studies, identification of microbes inhabiting a particular ecosystem, detection of biological warfare agents, detection of water- and food-borne pathogens and detection of blood and urinary tract pathogens, detection of antibiotic resistance etc. This review provides an overview of the status and recent applications of mass spectrometry (MS) for microbial identification and explores the usefulness of this technology for diagnosis of diseases caused by bacteria, viruses, and fungi.

**Table 1:** Microbial detection methods used in clinical microbiology.

Detection method	Advantages	Disadvantages
Conventional; culture on microbiological media and identification by biochemical tests	• Sensitive • Inexpensive	• Lengthy and time consuming process • Might require 24–48 h
Immunological-based methods	• Faster than conventional methods • Can detect both contaminating organisms and their toxins	• Not as specific, sensitive, and rapid as nucleic-acid based detection methods • Require large amounts of antigen • Developed for only a small number of microorganisms
Florescent *in situ* hybridization (FISH)	• Rapid detection and identification directly from slide smears • Fast and ease-of use of conventional staining methods combined with specificity of molecular methods	• Test limited by the availability of specific antigens for detection
Florescent *in situ* hybridization (FISH)	• Rapid detection and identification directly from slide smears • Fast and ease-of use of conventional staining methods combined with specificity of molecular methods	• Test limited by the availability of specific antigens for detection
Molecular based methods	(i) Real-time PCR (ii) Multiplex-PCR	• Culturing of the sample is not required • Specific, sensitive, rapid, and accurate • Closed-tube system reduces the risk of contamination • Can detect many pathogens simultaneously	• A highly precise thermal cycler is needed • Trained laboratory personnel required for performing the test
DNA sequencing	• 16S rDNA and 18S rDNA sequencing are the gold standards • Can identify fastidious and uncultivable microorganisms	• Trained laboratory personnel and powerful interpretation softwares are required • Expensive • Not suitable for routine clinical use
Microarrays	• Large scale screening system for simultaneous diagnosis and detection of many pathogens	• Trained laboratory personnel and powerful interpretation softwares are required • Expensive • Trained laboratory personnel required
Loop-mediated isothermal amplification (LAMP) assay	• Can generate large copies of DNA in less than an hour • No sophisticated equipment is required	• Trained laboratory personnel and powerful interpretation softwares are required • Expensive • Trained laboratory personnel required
Loop-mediated isothermal amplification (LAMP) assay	• Can generate large copies of DNA in less than an hour • Easy to use • No sophisticated equipment is required	• Developed for only a small number of microorganisms as yet
Metagenomic assay	• Useful for random detection of pathogens	• Data acquisition and data analysis is time consuming • Trained laboratory personnel required
MALDI-TOF MS	• Fast • Accurate • Less expensive than molecular and immunological-based detection methods • Trained laboratory personnel not required	• High initial cost of the MALDI-TOF equipment

### Mass Spectrometry

Mass spectrometry is an analytical technique in which chemical compounds are ionized into charged molecules and ratio of their mass to charge (m/z) is measured. Though MS was discovered in the early 1900s, its scope was limited to the chemical sciences. However, the development of electron spray ionization (ESI) and matrix assisted laser desorption ionization (MALDI) in 1980s increased the applicability of MS to large biological molecules like proteins. In both ESI and MALDI, peptides are converted into ions by either addition or loss of one or more than one protons. Both are based on “soft ionization” methods where ion formation does not lead to a significant loss of sample integrity. MALDI-TOF MS has certain advantages over ESI-MS viz. (i) MALDI-TOF MS produces singly charged ions, thus interpretation of data is easy comparative to ESI-MS, (ii) for analysis by ESI-MS, prior separation by chromatography is required which is not needed for MALDI-TOF MS analysis (Everley et al., 2008). Consequently, the high throughput and speed associated with complete automation has made MALDI-TOF mass spectrometer an obvious choice for proteomics work on large-scale (Ekström et al., 2000).

### MALDI – Principle and Methodology

**FIGURE 1:**
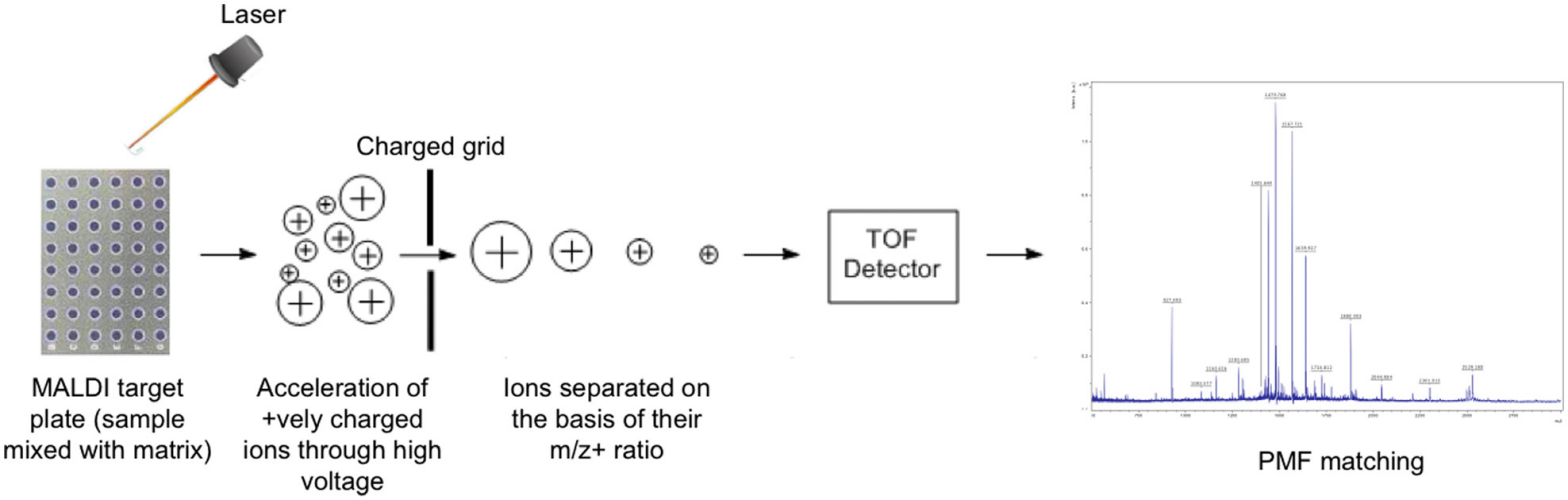
**Schematic diagram showing the work-flow in a MALDI-TOF MS**.

The sample for analysis by MALDI MS is prepared by mixing or coating with solution of an energy-absorbent, organic compound called matrix. When the matrix crystallizes on drying, the sample entrapped within the matrix also co-crystallizes. The sample within the matrix is ionized in an automated mode with a laser beam. Desorption and ionization with the laser beam generates singly protonated ions from analytes in the sample. The protonated ions are then accelerated at a fixed potential, where these separate from each other on the basis of their mass-to-charge ratio (m/z). The charged analytes are then detected and measured using different types of mass analyzers like quadrupole mass analyzers, ion trap analyzers, time of flight (TOF) analyzers etc. For microbiological applications mainly TOF mass analyzers are used. During MALDI-TOF analysis, the m/z ratio of an ion is measured by determining the time required for it to travel the length of the flight tube. A few TOF analyzers incorporate an ion mirror at the rear end of the flight tube, which serves to reflect back ions through the flight tube to a detector. Thus, the ion mirror not only increases the length of the flight tube, it also corrects small differences in energy among ions (Yates, 1998). Based on the TOF information, a characteristic spectrum called peptide mass fingerprint (PMF) is generated for analytes in the sample (**Figure 1**).

Identification of microbes by MALDI-TOF MS is done by either comparing the PMF of unknown organism with the PMFs contained in the database, or by matching the masses of biomarkers of unknown organism with the proteome database. In PMF matching, the MS spectrum of unknown microbial isolates is compared with the MS spectra of known microbial isolates contained in the database. For species level identification of microbes, a typical mass range m/z of 2–20 kDa is used, which represents mainly ribosomal proteins along with a few housekeeping proteins. The characteristic pattern of highly abundant ribosomal proteins, which represent about 60–70% of the dry weight of a microbial cell, in the mass range of 2–20 kDa (Murray, 2012) is used to identify a particular microorganism by matching its PMF pattern with the PMFs of the ribosomal proteins contained in an extensive open database. Thus, the identity of a microorganism can be established down to the genus, and in many cases to the species and strain level (Fagerquist et al., 2010). This approach is widely used in microbial identification because it is simple and can be conveniently adopted in a microbial diagnostic laboratory, aided by the availability of many commercial libraries of organism PMFs. Microbial identification by matching the biomarker masses with the molecular masses of proteins predicted from the genome sequence is not very popular in microbiological diagnostic laboratories because it requires knowledge of complete genome sequence of an organism before a database of its predicted protein molecular masses could be created.

Although, the culture conditions might profoundly affect the microbial physiology and protein expression profile (Welker, 2011) they do not influence microbial identification by MALDI-TOF MS. Valentine et al. (2005) cultured three bacterial species on four different culture media and found that microbial MALDI-TOF MS identification was independent of culture conditions. Another research group (Carbonnelle et al., 2007) also reported that both the culture conditions and the culture time did not affect microbial identification by MALDI-TOF MS.

A number of organic compounds have been used as matrices for MALDI-TOF MS but for microbiological applications, α-cyano-4-hydroxycinnamic acid (CHCA), 2,5-dihydroxy benzoic acid (DHB), and 3,5-dimethoxy-4-hydroxycinnamic acid (sinapinic acid) have been found to be the most useful. The matrix solution consists of water and a mixture of organic solvents containing ethanol/methanol or acetonitrile and a strong acid like trifluoro acetic acid (TFA), which dissolves the matrix. The solvents penetrate the cell wall of microorganisms and extract out the intracellular proteins. When the solvent evaporates, ‘co-crystallization’ of protein molecules and other cellular compounds entrapped within the matrix solution takes place (Horneffer et al., 2001).

The process of sample preparation for identification of microbes by MALDI-TOF MS depends upon the source from which it was isolated, or on the chemical nature of the constituents of its cell wall. Investigators have evaluated different sample preparation methods for different groups of microorganisms. Some microbes might be identified directly by MS, called direct cell profiling, while for some others whole cell lysates or crude cell extracts are prepared. In direct cell profiling, a single colony of microorganism is picked and spotted directly on to the sample plate and immediately overlaid with the matrix solution. Gram negative bacteria like *Neisseria* spp. (Ilina et al., 2009), *Yersinia* spp. (Stephan et al., 2011), and *Vibrio* spp. (Eddabra et al., 2012) were identified by MALDI-TOF MS using direct cell profiling. A ‘preparatory extraction’ of microbes with formic acid (FA) reportedly increased the ability of MALDI-TOF MS in identifying Gram-positive species. Studies have reported that preparatory extraction was necessary for identification of Gram-positive bacteria by MALDI-TOF MS, but not for Gram-negative bacteria (Alatoom et al., 2011; Saffert et al., 2011). A ‘preparatory extraction’ of microbes with formic acid was also used for sample preparation of sugar-non fermenting bacterial species (Mellmann et al., 2008) and *Staphylococcus* spp. (Dubois et al., 2010).

Due to the complex nature of their cell walls aerobic actinomycetes, *Nocardia* and mycobacteria require specialized processing procedures prior to MALDI-TOF analysis. Verroken et al. (2010) described a modified procedure for identification of *Nocardia* spp. by MALDI-TOF MS. Bacteria were lysed in boiling water, followed by ethanol precipitation of proteins. The precipitated proteins were dried, resuspended in 70% formic acid and acetonitrile and analyzed by MALDI-TOF MS. Researchers have reported different methods for sample preparation for identification of mycobacteria by MALDI-TOF MS, ranging from direct bacterial profiling to treatment with formic acid, safety being a major concern for routine investigations. EI Khéchine et al. (2011) described a procedure which combined inactivation and processing methods. Mycobacterial colonies collected in screw-cap tubes containing water and 0.5% Tween 20 were inactivated by heating at 95°C for 1 h. Inactivated samples were centrifuged and vortexed with glass beads to disrupt the mycobacterial cell wall, the resultant pellet was re suspended in formic acid, acetonitrile, and centrifuged again. Finally, the supernatant was deposited onto the MALDI target plate and overlaid with matrix.

As in bacteria, various sample processing methods have been investigated for identification of yeasts by MALDI-TOF MS, out of which ‘preparatory extraction’ with formic acid was reported to be most suitable (Stevenson et al., 2010b; Theel et al., 2012). Cassagne et al. (2014) evaluated five methods for sample preparation for fungal hyphae and spores. They finally devised a protocol wherein fungi were cultivated on Sabouraud gentamicin-chloramphenicol agar for 72 h at 27°C, extracted with formic acid following incubation in ethanol. Acetonitrile was added, the mixture was centrifuged and supernatant was used for MALDI-TOF MS analysis. Lau et al. (2013) reported a method based on mechanical lysis for sample preparation of fungal hyphae and spores. A small specimen from the mold isolate was suspended in ethanol and zirconia-silica beads, vortexed and centrifuged. The pellet was re suspended in 70% FA, vortexed again, and centrifuged. The supernatant was used for subsequent analysis by MALDI-TOF MS. Both methods reportedly gave good results in their respective studies. Analysis of intact cells of members of the genus *Penicillium* generated poor MALDI spectra, but re suspension of the conidia and spores in trifluoroacetic acid-acetonitrile and disruption with glass beads discriminated the species with 100% accuracy (Hettick et al., 2008a).

The discovery of suitable matrices, usage of whole/intact cells for recording PMF of microbes in the typical mass range (m/z) of 2–20 kDa, followed by availability of dedicated databases for microbial identification has made MALDI-TOF MS a lucrative alternative for microbial identification. The first MALDI-TOF MS system capable of microbial identification, termed “MALDI Biotyper” was developed by Bruker Daltonics. The MALDI Biotyper consisted of a basic MALDI-TOF platform, operating and analysis softwares, an onsite database and a simple method for extraction/preparation of sample (Seng et al., 2009). The software and database integrated easily with the Bruker MALDI-TOF instrument and its associated operating and analysis softwares. This system also allowed for up gradation by providing option of adding internal libraries of organisms. Another MALDI platform for microbial identification was introduced by Shimadzu in collaboration with bioMérieux. Shimadzu supplied the instrumentation and the analysis software “Launchpad,” while bioMérieux supplied and maintained the centralized database, “SARAMIS” which could be constantly updated. Later, Andromas introduced a different kind of database and software for routine bacteriological identification which was compatible with both Bruker and Shimadzu instruments (Emonet et al., 2010). The greatest break-through in the development of MALDI-TOF MS came with its regulatory approval for routine identification of bacteria and fungi in clinical microbiological laboratories. In the last 2 years, MALDI Biotyper CA System (Bruker Daltonics Inc.) has been approved by the US Food and Drug Administration (FDA) for identification of cultured bacteria from human specimens (*in vitro* diagnosis). Similarly, VITEK^®^ MS (bioMerieux Inc.) was approved by the US FDA for identification of cultured bacteria and yeasts (Patel, 2015). The list of microorganisms approved for identification is unique to the individual system (Patel, 2015). MALDI-TOF MS was first approved for clinical use in China in 2012 when bioMérieux VITEK MS system was approved by China State FDA for *in vitro* diagnostic (IVD) purposes (Luo et al., 2015). Later, China State FDA also approved the Bruker IVD MALDI Biotyper System for routine identification of microorganisms isolated from human specimens.

The organism databases are the key components of commercial MALDI platforms. They are continually increasing in size and are regularly updated by the manufacturers with discovery of new microbial species and annotations. Both, the Bruker and the Shimadzu systems contained a large collection of representative organisms in their databases and yielded comparable results with very low false positive rates (Carbonnelle et al., 2012). With a few exceptions, isolates were rarely misidentified by these devices. In a few MALDI-based identification studies, some organisms could not be identified; this failure was attributed to the organism not being included in the earlier databases rather than to the methodological error (Carbonnelle et al., 2012). A critical drawback of the proprietary softwares and databases is their limited accessibility to the private researchers due to their high costs. Nevertheless, a few research groups have developed some open-source softwares and databases which are freely available for the scientific community. To name a few, these are, mMASS, Mass-Up, pkDACLASS, MALDIquant, SpectraBank, BIOSPEAN, etc. (Strohalm et al., 2008; Ndukum et al., 2011; Böhme et al., 2012; Gibb and Strimmer, 2012; Raus and Šebela, 2013).

## Applications in Microbial Diagnosis

### MALDI-TOF MS in Bacteriology

#### Clinical Bacteriology

Conventionally diagnosis of bacterial infections in the body fluids is carried out on the basis of biochemical and metabolic-profiling that requires 24–48 h for identification of the inflicting bacterial species. In the meantime, patients are administered empirical antibiotics, which are sometimes inappropriate. Clinical microbiology laboratories require rapid, reliable, and cost effective methods for identification of potential pathogens in clinical samples so that appropriate antimicrobial therapy may be initiated early. A number of researchers have shown that MALDI-TOF MS can be used for early identification of bacteria in blood cultures, urinary tract infections (UTIs), cerebrospinal fluids, respiratory tract infections, stool samples etc.

Many studies have shown that MALDI-TOF MS equalled or even surpassed the conventional diagnostic methods in speed and accuracy in detecting blood stream infections (La Scola and Raoult, 2009; Stevenson et al., 2010a; Foster, 2013; Haigh et al., 2013; Tadros and Petrich, 2013). A few studies suggested that additional pre-treatment of body fluids by ammonium chloride (Prod’hom et al., 2010), formic acid (Christner et al., 2010), or short-term incubation on solid medium (Idelevich et al., 2014) further improved the diagnostic potential of MALDI-TOF MS.

When conventional methods for identification of urinary tract pathogens for diagnosis of UTIs were compared with MALDI-TOF MS based identification systems, it was found that MALDI-TOF MS required minimal processing time and identified bacteria from urine samples in the presence of even more than two uropathogens (Ferreira et al., 2010; Köhling et al., 2012; Burillo et al., 2014). A few researchers have suggested procedures involving differential centrifugation of urine samples (Rosselló et al., 2014) or diafiltration (Demarco and Burnham, 2014) to further improve the clinical sensitivity and turnaround time of MALDI-TOF MS based diagnosis of UTI.

Diagnosis of infectious diarrhea in laboratory is usually done by culture and identification of bacteria in the stool samples. This is a costly and time consuming process requiring 3–5 days for detection and identification of enteric bacterial pathogens. He et al. (2010) performed a comparative study of identification by MALDI-TOF MS versus routine phenotypic methods for identification of suspicious colonies from stool samples. They found that the entire procedure for identification by MALDI-TOF MS, from smear preparation to reporting of the final result was completed within 30 min, thus shortening the turnaround time of the test by 2–3 days.

Bacterial meningitis is a neurological emergency. Early diagnosis is vital for rapid initiation of appropriate antimicrobial therapy. MALDI-TOF MS has been used for direct detection of bacteria causing meningitis in cerebrospinal fluids (Segawa et al., 2014). It has also been used for rapid identification of atypical, Gram-negative environmental organisms and respiratory tract pathogens which chronically infect patients with cystic fibrosis (Alby et al., 2013; Baillie et al., 2013). Recently, a very novel application of MALDI-TOF MS was shown by Guembe et al. (2014) who reported that MALDI-TOF MS can perform better than conventional culture methods in diagnosis of catheter-related bloodstream infections.

### Food- and Water-Borne Bacteria

Rapid identification of pathogenic microorganisms is important to ensure safety and quality of water and food products. MALDI-TOF MS has been shown to be useful for early detection of bacterial hazards which might contaminate drinking water. The genus *Aeromonas* which is indigenous to surface waters is currently composed of 17 species, of which seven can cause severe water-borne outbreaks. Donohue et al. (2007) used the m/z signature of known strains of *Aeromonas* to assign species to unknown environmental isolates. Their results showed that MALDI-TOF MS rapidly and accurately classified unknown species of the genus *Aeromonas*, which was suitable for environmental monitoring.

MALDI-TOF MS has also been applied successfully in food microbiology for various purposes like, identification and classification of lactic acid bacteria in fermented food (Nguyen et al., 2013), detection of bacteria involved in spoilage of milk and pork (Nicolaou et al., 2012), identification of bacteria isolated from milk of dairy cows (Barreiro et al., 2010), identification of bacteria present in probiotics and yogurt (Angelakis et al., 2011), identification of pathogenic bacteria contaminating powdered infant formula-food (Stephan et al., 2010), characterization of biogenic amine-producing bacteria responsible for food poisoning (Fernández-No et al., 2010) and for identification of causative agents of seafood-borne bacterial gastroenteritis (Hazen et al., 2009; Böhme et al., 2010, 2011).

### Environmental Bacteriology

Tests based on biochemical traits usually fail to identify microbes isolated from environmental samples, as the diversity of microbes in these habitats is enormous (Torsvik et al., 2002). Various studies have shown that whole cell MALDI-TOF MS can be used as an efficient tool to identify and characterize isolates which originate from specific ecosystems. Researchers have reported the use of MALDI-TOF MS in identification of microbes isolated from sewage sludge (Ruelle et al., 2004), for grouping of bacteria isolated from marine sponges into different proteotaxanomic groups (Dieckmann et al., 2005) and for identifying bacteria inhabiting soil contaminated with polychlorinated biphenyl (Uhlik et al., 2011).

Another research group performed MALDI-TOF MS analysis of the cultivable fraction of environmental samples. They acquired strain-specific spectra which helped in grouping aerobic and moderately halophilic prokaryotes into phenotypic clusters belonging to distinct taxa. They suggested that, if the culture conditions were not radically different, irrespective of the age or quality of the culture, MS spectrum of a microbe reflects its taxon-specific phenotypic properties which results in guaranteed identification (Munoz et al., 2011).

In another study MALDI-TOF MS was used for differentiating bacterial species of the family Rhizobiaceae. Subdivided within three genera, these bacterial species establish either symbiotic or saprophytic interactions with plants.The research group even constructed a database that included the type strains of currently accepted species in the family and validated it by identifying all rhizobial strains isolated from nodules and tumors (Ferreira et al., 2011).

### Detection and Identification of Agents of Biological Warfare

Fast and reliable identification of microbes which pose threats as agents of bioterrorism is required, not only to combat biological-warfare attacks, but also to prevent natural outbreaks caused by these organisms. Conventionally, organisms which pose severe threats as agents of bioterrorism have been identified by phenotypic, genotypic, and immunological identification systems which are slow, cumbersome and pose significant risk to the laboratory personnel. Recently various researchers reported MALDI-TOF MS as a simple, rapid and reliable approach to identify highly pathogenic organisms like *Brucella* spp., *Coxiella burnetti*, *Bacillus anthracis, Francisella tularensis*, and *Y. pestis* (Shaw et al., 2004; Pierce et al., 2007; Lasch et al., 2009; Ayyadurai et al., 2010; Seibold et al., 2010; Lista et al., 2011; Vranakis et al., 2013).

Further work is being carried out to develop safe and MS-compatible protocols for inactivation of vegetative cells and spores of highly pathogenic organisms, which can be integrated into a routine microbiological laboratory. Lasch et al. (2008) proposed a tri-fluoro acetic acid (TFA) based inactivation protocol for vegetative cells and spores of pathogenic organisms, but Couderc et al. (2012) found that for *Yersinia* isolates, ethanol inactivation yielded MALDI-TOF MS spectra of significantly higher quality than TFA inactivation. Jeong et al. (2014) reported a direct *in situ* MALDI-TOF MS which allowed a high-throughput detection and identification of aerosolized *Bacillus* spores, without any pretreatment process. The spores of the *Bacillus* spp. were directly spotted on the MALDI target plate and left for air-drying. These were subsequently coated with the matrix solution. After the matrix solution air-dried, the spotted samples were analyzed by MS (Jeong et al., 2013, 2014).

MALDI-TOF MS has also been shown to be useful for detection of protein toxins, such as staphylococcal enterotoxin B, botulinum neurotoxins, *Clostridium perfringens* epsilon toxin, shiga toxin etc. which can be used as potential agents for bioterrorism when delivered via an aerosol route (Kull et al., 2010; Alam et al., 2012). In a biological war, early and unambiguous detection of toxins from aerosols is required to initiate medical countermeasures. Alam et al. (2012) developed a simple method of sample processing for identification of protein toxins by MALDI-TOF/TOF MS method. Nebulizer was used to generate aerosols which were collected using a cyclone collector. Tandem MS data with information from peptide sequences was used for detecting toxins that originated from organisms of any geographical location.

### Detection of Antibiotic Resistance in Bacteria

MALDI-TOF MS has been shown to generate PMFs capable of discriminating lineages of methicillin-resistant *S. aureus* strains (Wolters et al., 2011). Under careful experimental conditions, it has also been shown useful for subtyping methicillin-resistant *S. aureus* strains (Croxatto et al., 2012). Similarly, MALDI-TOF MS has been shown to be of great use in identifying vancomycin-resistant enterococci (Griffin et al., 2012; Nakano et al., 2014). Wang et al. (2014) suggested that MALDI-TOF MS could be used as a screening tool for discriminating vancomycin-resistant *Enterococcus faecium* strains from vancomycin-susceptible *E. faecium* strains.

The most common mode of microbial resistance to β-lactams, the largest class of antibiotics, is their enzymatic hydrolysis by β-lactamases. The production of β-lactamases is detected by MALDI-TOF MS employing a ‘mass spectrometric β-lactamase (MSBL) assay.’ In this assay, buffered solution of the antibiotic is mixed with bacterial culture and incubated. The reaction mixture is centrifuged and supernatant subjected to MALDI-TOF MS analysis. The β-lactamase producers inactivate the β-lactam ring of the antibiotic by addition of a residue of water. The mass shift in the non-hydrolyzed and the hydrolyzed forms of the antibiotic confirms the presence or absence of β-lactamase producing bacteria. The MSBL assay has been applied for detection of resistance to β-lactam antibiotics like penicillin, ampicillin, piperacillin, cetazidime, cefotaxime, ertapenem, meropenem, and imipenem. Using MSBL assay researchers have successfully detected β-lactamase producing organisms like *Escherichia coli, Klebsiella pneumoniae, Pseudomonas aeruginosa, Acinetobacter baumanni, Citrobacter freundii, Enterobacter cloaceae, Salmonalla* spp. etc. (Hrabák et al., 2011; Hooff et al., 2012; Sparbier et al., 2012a; Kostrzewa et al., 2013). Given the accuracy of resistance detection with MSBLs, further innovations and improvements in terms of enhancing speed of resistance detection and expanding detection of resistance for newer classes of β-lactam antibiotics in MSBL assays are being envisaged. Recently, Johansson et al. (2014) developed a MALDI-TOF MS method for detection and verification of carbapenemase production in anaerobic bacterium, *Bacteroides fragilis* as early as 2.5 h. Hoyos-Mallecot et al. (2014) evaluated a MALDI-TOF MS method for identification and differentiation of carbapenemase producing clinical strains of *Enterobacteriaceae* and *P. aeruginosa* from metallo-β-lactamase producing strains. Hart et al. (2015) reported a modified strategy for detection of biomarkers of antibiotic resistance in clinical strains of *E. coli* using MALDI-TOF MS. They suggested that instead of using intact bacterial cells for MS, the periplasmic compartment should be extracted (since β-lactamases are located in the periplasm); in-solution digested with trypsin, separated by nano-LC before MALDI-TOF MS analysis. Using this approach they reported the peptide sequence of biomarkers for several classes of β-lactamases like CTX-M-1 group extended spectrum β-lactamase, TEM β-lactamase, VIM a metallo-β-lactamase and CMY-2 an ampC β-lactamase. Moreover using this approach they also detected the peptides specific to an aminoglycoside modifying enzyme Kan-R.

Similar to β-lactams, resistance in microorganisms to aminoglycosides is mainly due to the enzymatic modification of antibiotics by bacterial acyltransferases, adenyltransferases, and phosphotransferases. Since these enzymes show different preferentiality for the CoA substrate present in different aminoglycosides, it has not been possible to establish a universal MS-based assay for detection of aminoglycoside resistance. Further efforts to develop and standardize MALDI-TOF MS for detection of aminoglycoside resistance in routine laboratory are still underway.

### Bacterial Strain Typing and Taxonomy

Conventional classification of bacteria has been carried out on the basis of biochemical, metabolic and antigenic properties. However, currently microbial species are being identified primarily on the basis of genomic information. The sequencing of 16S rDNA is considered to be the ‘gold standard,’ because it is present in every prokaryote and allows reconstruction of a global phylogeny (Woese et al., 1990; Stackebrandt and Goebel, 1994; Pace, 1997). Most DNA fingerprinting methods, however, do not correlate phylogenetically and fail to give any information regarding the evolutionary relationship between various species. Genomic techniques like amplified fragment length polymorphism (AFLP), pulsed-field gel electrophoresis (PFGE), and multilocus sequence typing (MLST) are used for subtyping of bacteria for epidemiological purposes. Though 16S rDNA sequencing is adequate to assign genus or species to an isolate, in most cases it is not sufficient for refined typing, e.g., epidemiological studies. PFGE, can be applied for high resolution typing, but is generally inappropriate for assigning genus or species to microorganisms (O’Leary et al., 2011).

Proteomics represents the functional aspect of genomics and can be used as a taxonomic tool. Gel-based whole cell protein profiling may be as cumbersome and time consuming as any other genomic technique. On the other hand MALDI-TOF MS intact cell or whole cell PMF based typing is a rapid and sensitive method for bacterial identification. In many cases it has shown resolution and reproducibility which is better than gel-based protein or DNA fingerprinting techniques (van Baar, 2000; Fenselau and Demirev, 2001; Lay, 2001).

Numerous studies have shown that MALDI-TOF MS is a rapid, reliable and cost-effective technique for identification of bacteria. The method, however, has some lacunae: (i) proper identification of organisms is possible only when the spectral database contains information about specific genes like prokaryotic 16S rRNA, *gyr*B, *rpo*B, or *hsp*60 of strains/species of a particular genus, (ii) databases should be prepared locally for certain taxa (e.g., *Streptococcus* or *Staphylococcus*) in which geographical variations lead to variations in the genotype and phenotype (Benagli et al., 2011).

During the past few years various researchers have shown applicability of MS for bacterial identification, taxonomy and strain typing. Haag et al. (1998) used MALDI-TOF MS for rapid characterization of pathogenic *Haemophilus* strains. They also determined strain-specific differences among *H. influenzae* isolates from several patients. Nilsson (1999), detected strain-specific biomarkers based on the analysis of six different strains of *Helicobacter pylori*. Lundquist et al. (2005) showed differentiation of four subspecies of *F. tularensis* using this approach. Carbonnelle et al. (2007) showed that MALDI-TOF MS was a powerful tool for the identification of clinical isolates of coagulase negative staphylococci. MALDI-TOF MS was used for rapid identification of ten different species of *S. viridans* (Friedrichs et al., 2007). When the results of species identification obtained by MALDI-TOF MS were compared with the phenotypic/genotypic identification systems, a 100% consonance was achieved. Similarly, a variety of *Staphylococcus* spp., pathogenic *Neisseria* spp., clinically important Yeast species and *Mycobacterium* spp. were identified using MALDI-TOF MS (Ilina et al., 2009; Dubois et al., 2010; Stevenson et al., 2010b; Saleeb et al., 2011). **Table 2** lists microorganisms in which intact cell (direct bacterial profiling) was used for bacterial identification and strain typing.

**Table 2:** Bacteria in which MALDI-TOF MS was used for identification and strain typing.

Organisms	Reference
*Acinetobacter* spp.	Espinal et al. (2011), Alvarez-Buylla et al. (2012)
*Aeromonas* spp.	Donohue et al. (2006, 2007), Lamy et al. (2011)
*Arcanobacterium* spp., *Arcanobacterium haemolyticum*	Hijazin et al. (2011), Vila et al. (2012)
*Bacteroides* spp.	Nagy et al. (2009)
β-hemolytic streptococci	Cherkaoui et al. (2011)
*Brucella* spp.	Lista et al. (2011)
*Campylobacter* spp.	Kiehntopf et al. (2011)
*Cardiobacterium hominis endocarditis*	Wallet et al. (2011)
*Clavibacter* spp.	Zaluga et al. (2011)
*Corynebacterium* spp.	Alatoom et al. (2012), Vila et al. (2012)
*Cronobacter* spp.	Zhu et al. (2011)
*Francisella tularensis*	Seibold et al. (2010)
*Ochrobactrum anthropic*	Quirino et al. (2014)
*Escherichia* strains	Muroi et al. (2011)
*Gallibacterium* spp.	Alispahic et al. (2011)
*Haemophilus* spp.	Nørskov-Lauritsen et al. (2012)
*Helicobacter pylori*	Ilina et al. (2010)
*Legionella* spp.	He et al. (2011)
*Mycobacterium* spp.	Hettick et al. (2004), Lefmann et al. (2004), Saleeb et al. (2011), Wang et al. (2012)
*Neisseria* spp.	Ilina et al. (2009)
Members of family *Pasteurellaceae*	Kuhnert et al. (2012)
*Rhodococcus equi*	Vila et al. (2012)
*Salmonella* spp.	Wang et al. (2008), Dieckmann and Malorny (2011), Sparbier et al. (2012b)

MALDI-TOF MS is not only used for species identification but has application in strain typing also. Kumar et al. (2004) showed that MALDI-TOF MS may be a useful tool for discriminating strains of beta hemolytic streptococci, and also for characterization of untypable strains of streptococci group A. Eddabra et al. (2012) used MALDI-TOF MS to discriminate 30 environmental strains of *Vibrio* spp. Stephan et al. (2011) used MALDI-TOF MS for rapid identification of *Y. enterocolitica* strains to the species, and subtyping to the biotype level. MALDI-TOF MS based methods for the discrimination and typing of mycobacteria, typing multidrug-resistant *K. pneumonia*, and typing strains responsible for nosocomial outbreak of *A. baumannii* have been described (Shitikov et al., 2012; Berrazeg et al., 2013; Mencacci et al., 2013).

MALDI-TOF MS spectrum of an individual microbe is the taxon-specific property of that organism, which is independent of its geographical location, culture conditions (which should not be drastically different) or sample preparation methodology. With this approach, not only identification of new isolates as members of existing species is possible if their type strains have been previously studied (Ruelle et al., 2004), recognition of coherent phenotypic patterns reflecting taxonomic identities is also possible. The ease and rapidity in identification of large numbers of isolates can be used to study taxonomic and inter- and intra-specific diversity (Feli and Dellaglio, 2007).

## MALDI-TOF MS in Virology

### Clinical Virology

Viruses were traditionally detected by cell culture, which in spite of being the gold standard, often took days or even weeks before any results were available. It was later replaced or complemented by lesser sensitive immunological methods, based on antibody analysis (by immunoassays or immunofluorescence) and by more sensitive molecular methods based on PCR and dot blot hybridization. The use of MALDI-TOF MS in virology has advanced less as it has in bacteriology or mycology. This might be a consequence of the relatively low protein content of viruses (Kliem and Sauer, 2012), higher molecular weight of viral proteins (>20,000 Da) and a probable carryover of debris of the cell substrate in which viruses are cultured *in vitro*. Nevertheless, many researchers have proved the utility of MALDI-TOF MS for diagnosis of various infectious viruses in clinical samples like influenza viruses, enteroviruses, human papilloma viruses (HPVs), herpes virus, hepatitis virus etc. (Sjöholm et al., 2008; Yi et al., 2011; Piao et al., 2012). Interestingly in most of the studies, the viral genetic material was amplified by PCR and the amplicons were analyzed/identified by MALDI. Sjöholm et al. (2008) developed an efficient MALDI-TOF MS based screening method for multiplex detection of all human herpes viruses which were present in different archival biological samples. The sensitivity and the detection limit of viruses by MALDI-TOF MS method was high and comparable to reference methods like oligonucleotide microarrays and multiplex PCR (Sjöholm et al., 2008).

Yi et al. (2011) reported the use of a PCR-based MS method for detection of high-risk HPVs, a prime cause of human cervical cancer. They claimed that the high-throughput and cost-effectiveness associated with the method made it suitable for diagnosis in routine clinical settings and for epidemiological studies. Piao et al. (2012) combined the multiplex PCR with MALDI-TOF MS and developed a PCR-Mass assay which simultaneously detected eight distinct viruses associated with enteric infections in humans. Later, Peng et al. (2013) proved the utility of multiplexed MALDI-TOF for type-specific detection of human enteroviruses associated with hand, foot and mouth diseases. Recently, Calderaro et al. (2014) reported that MALDI-TOF MS was an effective, rapid and inexpensive tool which identified various poliovirus serotypes from different clinical samples. They further reported that through MALDI-TOF MS, specific viral biomarkers could be detected which were helpful in differentiating virus-infected cells from healthy cells.

### Viral Genotyping, Subtyping, and Epidemiological Studies

Apart from viral identification, MALDI-TOF MS has also been used in virology for genotyping of JC polyomaviruses (Bayliss et al., 2010), hepatitis B and hepatitis C viruses (Ilina et al., 2005; Ganova-Raeva et al., 2010) and for detection of mutations in hepatitis B viruses (Luan et al., 2009). Also, many researchers have demonstrated the application of MALDI-TOF MS for screening of influenza virus subtypes and for tracking epidemiology of influenza viruses (Schwahn et al., 2009, 2010; Fernandes and Downard, 2014). Newer strains of influenza viruses frequently originate by mixing of genetic material of several strains in a common host. Thus, detection of ever evolving influenza viruses is a challenge for PCR based molecular methods, because the primers fail to anneal to their respective target sequences and need to be redesigned repeatedly. Similarly, the antibody-based immunological assays fail to identify the antigenically distinct, reassorted virus strains. Rapid identification and characterization of the inflicting influenza viral strains is necessary to initiate an early, effective therapy and to prevent a probable pandemic. MALDI-TOF MS has emerged as a rapid and reliable approach to screen highly evolving influenza virus types and subtypes. Chou et al. (2011) reported the use of MALDI-TOF MS in combination with antibody-magnetic nanoparticles for detection and rapid screening of influenza virus subtypes. Later, Downard (2013) described a method for detection of strains of influenza viruses using whole virus protein digests. This ‘proteotyping approach’ was successful in typing, subtyping, and tracing the lineage of human influenza viruses. He even described the success of his ‘proteotyping approach’ in characterization of strains of parainfluenza virus, another respiratory infectious agent.

### Detection of Antiviral Resistance

The studies in this area are scanty. However, MALDI-TOF MS has proved efficacy in detecting drug resistance to ganciclovir in cytomegaloviruses which frequently infect transplant recipients (Zürcher et al., 2012).

## MALDI-TOF MS in Mycology

### Clinical Mycology

Conventional methods for identification of fungi are based on morphological, biochemical, and/or immunological properties which might span 2–5 days, or more, and often require combining several phenotypic methods for conclusive interpretations. The molecular methods based on analysis of genes encoding 18S rRNA and the internal transcribed spacer regions 1 and/or 2 (ITS 1/2) are tedious and time consuming (Sendid et al., 2013). Rapid and accurate identification of the inflicting fungal species is required for early initiation of antifungal therapy.

Fungal identification by MALDI-TOF MS in the medical mycology laboratory has moved at a slower pace than bacterial identification, owing to their inherent biological complexity which makes their study as a whole difficult, and also due to co-existence of different fungal phenotypes (hyphae and/or conidia) in the same organism (Santos et al., 2010). In order to obtain reproducible PMF results, parameters like culture media, quantity/type of colony material and incubation time, need to be carefully standardized. Also, the fungal cells might require additional treatment with trifluoroacetic acid, formic acid, or acetonitrile along with beating with beads to disrupt strong cell walls.

The first study describing the use of MALDI-TOF MS for identification and characterization of single-celled fungus, *Saccharomyces cerevisiae* was reported in Amiri-Eliasi and Fenselau (2001). Among fungi, highly reproducible PMF spectra have been reported for the ascomycetous and basidiomycetous yeasts including organisms like *Candida*, *Cryptococcus*, and *Pichia*. These genera of yeast form uniform colonies on agar plates which can be lysed efficiently with the standard procedure (as used in bacteria) for MALDI-TOF sample preparation (Bader, 2013). However, Cassagne et al. (2014) evaluated various pretreatment procedures for MALDI-TOF MS identification of yeasts and reported that although labor-intensive, complete extraction with formic acid/acetonitrile yields better identification results. Various researchers have reported that MALDI-TOF MS was a reliable and time-saving approach for identification of various yeast species in bloodstream infections (Spanu et al., 2012; Yaman et al., 2012; Lavergne et al., 2013). Several researchers have described the use of MALDI-TOF MS for detection of various human fungal pathogens. These have been summarized in the **Table 3**.

**Table 3:** Fungi which have been identified using MALDI-TOF MS.

Organisms	Reference
*Fusarium* spp.	Dong et al. (2009), Kemptner et al. (2009)
*Aspergillus* spp.	Li et al. (2000), Hettick et al. (2008b), Alanio et al. (2011), Pan et al. (2011)
*Penicillium* spp.	Chen and Chen (2005)
*Fusarium proliferatum*	Seyfarth et al. (2008)
*Lichtheimia* spp.	Schröd et al. (2012)
Dermatophyte species	Erhard et al. (2008), Theel et al. (2011), Nenoff et al. (2013)
*Cryptococcus neoformans, C. gattii*	Mc Taggart et al. (2011)
*Neoscytalidium* spp.	Alshawa et al. (2012)
*Candida* spp.	Spanu et al. (2012)

### Detection of Antibiotic Resistance in Fungi

Identification/prediction of resistance for antifungals by MALDI-TOF MS has not advanced as much asSit has, in predicting resistance in bacteria. This might be attributed to the fact that antimycotic resistance in fungi is not as frequent as in bacteria due to absence of drug degrading enzymes. Only a few fungal species like *C. glabrata* or *C. krusei* and *C. parapsilosisis* have been reported to be intrinsically resistant to azoles and echinocandins respectively (Bader, 2013). Similarly, a species-specific resistance to antimycotic agents is observed in many molds and zygomycetes (Pfaller et al., 2002; Alastruey-Izquierdo et al., 2010). Thus, drug resistance in fungal isolates may be predicted simply by identification of the inflicting fungal species by MALDI-TOF MS (Bader, 2013).

### Fungal Strain Typing and Taxonomy

The use of MALDI-TOF MS for strain typing of fungal isolates is still in infancy and not as successful as in bacteria. In contrast to yeasts, it has been difficult to type molds since they have complicated phylogenetic relationships (Samson and Varga, 2009) and more complicated morphology. Strain typing by MALDI-TOF MS was, however, reported to be feasible with *C. albicans* and *C. parapsilosis* (Qian et al., 2008; Pulcrano et al., 2012).

## Future Perspectives

Although attempts for MS based bacterial identification and diagnosis date back to mid 1970s it is only in the recent years that microbiologists have realized the potential and applicability of MALDI-TOF MS in routine microbiological laboratories. The current gold standard for microbial identification – 16S rRNA and 18S rRNA gene sequencing is not favorable in terms of both cost and time. Bizzini et al. (2011) claimed that including the cost of consumables, salaries, and apparatus-depreciation, identification of a single bacterial isolate by 16S rRNA sequencing costed approximately 100 US dollars in their laboratory and the results were available after 48 h. On the other hand identification of a single bacterial isolate by MALDI-TOF MS could be done in minutes and costed only a few US dollars (Seng et al., 2009; Cherkaoui et al., 2010).

Comparison of PMF of unknown isolate to reference mass fingerprints present in the database is the most crucial step for species identification which requires a database containing not only reference mass fingerprints of all species of interest, but also mass fingerprints of multiple strains of each species (Lartigue et al., 2009). The MALDI-TOF MS technology failed to distinguish *E. coli* and *Shigella* spp. (Bizzini et al., 2010) or discriminate among species of the mitis complex of Streptococci (Seng et al., 2009; van Veen et al., 2010). The former failure has been explained by the fact that *E. coli* and *Shigella* were one species phylogenetically but due to historical and clinical constraints microbiologists have classified them into two species. The reason for improper delineation of *S. pneumoniae* and *S. mitis* is the close relationship between the two species (a microbiological constraint) which cannot be distinguished by any database update, except tests like optochin sensitivity or bile solubility (Kilian et al., 2008). Such constraints of MALDI-TOF MS certainly need to be addressed in future.

The major constraint against using MALDI-TOF MS in routine microbiological diagnosis is the reproducibility of the PMFs of the same microbial species during different experiments in the same laboratory or during different experiments in different laboratories employing the same/different MALDI-TOF equipment. The intra-laboratory reproducibility studies have shown a higher level of concordance in PMFs during repeated experiments with the same MS equipment using similar sample preparation technique (Walker et al., 2002). Another study reported that intra-laboratory reproducibility of PMFs depended only on the quality of the microbial samples and was independent of the sample preparation technique (Croxatto et al., 2012). However, studies on reproducibility of PMFs during inter-laboratory studies have yielded conflicting results. Two independent research studies showed a higher level of reproducibility in PMFs of identical microorganisms identified in separate laboratories employing similar sample preparation technique, the same MS equipment and the same analysis software (Wang et al., 1998; Walker et al., 2002). On the other hand, another study reported poor inter-laboratory concordance during MALDI-TOF analysis of identical aliquots of *E. coli* by three separate laboratories using similar sample preparation techniques, but different commercial MALDI-TOF equipment (Wunschel et al., 2005). This research group further combined the PMF spectra of individual laboratories and formed a ‘multiple laboratory master PMF.’ When this multiple laboratories’ master PMF spectra were used to identify *E. coli*, 100 percent identification was observed in each laboratory (Wunschel et al., 2005). Thus, this study provided a solution to a very important question that is, the potential of MALDI-TOF MS for microbial identification in different geographical locations with different MALDI-TOF equipment. By employing a standard protocol for MALDI-TOF MS analysis and creating a library/database containing PMFs from multiple laboratories (with different MALDI-TOF equipment), this constraint could be overcome easily.

In recent years there has been a plethora of information about MALDI-TOF MS and its application to a broad spectrum of microbes ranging from Gram positive to Gram negative bacteria, from clinical samples to extreme halophiles and from BSL-3 organisms to environmental isolates. Beyond the realms of microbial world, recent studies indicate that MALDI-TOF MS can even be applied to identify algae (von Bergen et al., 2009), mosquitoes (Yssouf et al., 2014), nematodes (Perera et al., 2005), and insects (Feltens et al., 2010; Hoppenheit et al., 2013). Independence of the culture conditions, culture formulations, cultivation time, quantity of inoculum required for identification, have made MALDI-TOF MS as the technique of choice in routine microbiological laboratories. The provision for integrating in-built databases in the public databases of MALDI-TOF MS, as well as development of inexpensive and user-friendly softwares for comparisons and analyses would further increase the credibility of MALDI-TOF MS in future. What seemed an exaggeration sometimes back has now become a reality. MALDI-TOF MS has become a valuable tool for a microbiological laboratory, which might potentially replace molecular identification techniques in near future.

## Conflict of Interest Statement

The authors declare that the research was conducted in the absence of any commercial or financial relationships that could be construed as a potential conflict of interest.

## References

[B1] Alam S. I. Kumar B. Kamboj D. V. (2012). Multiplex detection of protein toxins using MALDI-TOF-TOF tandem mass spectrometry: application in unambiguous toxin detection from bioaerosol. *Anal. Chem.* 84 10500–10507. 10.1021/ac3028678 23083074

[B2] Alanio A. Beretti J. L. Dauphin B. Mellado E. Quesne G. Lacroix C. (2011). Matrix-assisted laser desorption ionization time-of-flight mass spectrometry for fast and accurate identification of clinically relevant *Aspergillus* species. *Clin. Microbiol. Infect.* 17 750–755. 10.1111/j.1469-0691.2010.03323.x 20673266

[B3] Alastruey-Izquierdo A. Cuesta I. Walther G. Cuenca-Estrella M. Rodriguez-Tudela J. L. (2010). Antifungal susceptibility profile of human-pathogenic species of *Lichtheimia*. *Antimicrob. Agents Chemother.* 54 3058–3060. 10.1128/AAC.01270-09 20421405 PMC2897292

[B4] Alatoom A. A. Cazanave C. J. Cunningham S. A. Ihde S. M. Patel R. (2012). Identification of non-diphtheriae *Corynebacterium* by use of matrix-assisted laser desorption ionization-time of flight mass spectrometry. *J. Clin. Microbiol.* 50 160–163. 10.1128/JCM.05889-11 22075579 PMC3256690

[B5] Alatoom A. A. Cunningham S. A. Ihde S. M. Mandrekar J. Patel R. (2011). Comparison of direct colony method versus extraction method for identification of Gram-Positive cocci by use of bruker biotyper matrix-assisted laser desorption ionization–time of flight mass spectrometry. *J. Clin. Microbiol.* 49 2868–2873. 10.1128/JCM.00506-11 21613431 PMC3147777

[B6] Alby K. Gilligan P. H. Miller M. B. (2013). Comparison of matrix-assisted laser desorption ionization-time of flight (maldi-tof) mass spectrometry platforms for the identification of gram-negative rods from patients with cystic fibrosis. *J. Clin. Microbiol.* 51 3852–3854. 10.1128/JCM.01618-13 23966494 PMC3889780

[B7] Alispahic M. Christensen H. Hess C. Razzazi-Fazeli E. Bisgaard M. Hess M. (2011). Identification of Gallibacterium species by matrix-assisted laser desorption/ionization time-of-flight mass spectrometry evaluated by multilocus sequence analysis. *Int. J. Med. Microbiol.* 301 513–522. 10.1016/j.ijmm.2011.03.001 21596619

[B8] Alshawa K. Beretti J. L. Lacroix C. Feuilhade M. Dauphin B. Quesne G. (2012). Identification of clinical dermatophyte and *Neoscytalidium* species by matrix-assisted laser desorption ionization-time of flight mass spectrometry. *J. Clin. Microbiol.* 50 2277–2281. 10.1128/JCM.06634-11 22535981 PMC3405581

[B9] Alvarez-Buylla A. Culebras E. Picazo J. J. (2012). Identification of *Acinetobacter* species: is Bruker biotyper MALDI-TOF mass spectrometry a good alternative to molecular techniques? *Infect. Genet. Evol.* 12 345–349. 10.1016/j.meegid.2012.01.002 22266021

[B10] Amiri-Eliasi B. Fenselau C. (2001). Characterization of protein biomarkers desorbed by MALDI from whole fungal cells. *Anal. Chem.* 73 5228–5231. 10.1021/ac010651t 11721923

[B11] Angelakis E. Million M. Henry M. Raoult D. (2011). Rapid and accurate bacterial identification in probiotics and yoghurts by MALDI-TOF mass spectrometry. *J. Food Sci.* 76 M568–M571. 10.1111/j.1750-3841.2011.02369.x 22417598

[B12] Ayyadurai S. Flaudrops C. Raoult D. Drancourt M. (2010). Rapid identification and typing of *Yersinia pestis* and other *Yersinia* species by matrix-assisted laser desorption/ionization time-of-flight (MALDI-TOF) mass spectrometry. *BMC Microbiol.* 10:285 10.1186/1471-2180-10-285 PMC2992509 21073689

[B13] Bader O. (2013). MALDI-TOF-MS-based species identification and typing approaches in medical mycology. *Proteomics* 13 788–799. 10.1002/pmic.201200468 23281257

[B14] Baillie S. Ireland K. Warwick S. Wareham D. Wilks M. (2013). Matrix-assisted laser desorption/ionisation-time of flight mass spectrometry: rapid identification of bacteria isolated from patients with cystic fibrosis. *Br. J. Biomed. Sci.* 70 144–148. 24400425 10.1080/09674845.2013.11669948

[B15] Barreiro J. R. Ferreira C. R. Sanvido G. B. Kostrzewa M. Maier T. Wegemann B. (2010). Identification of subclinical cow mastitis pathogens in milk by matrix-assisted laser desorption/ionization time-of-flight mass spectrometry. *J. Dairy Sci.* 93 5661–5667. 10.3168/jds.2010-3614 21094737

[B16] Bayliss J. Moser R. Bowden S. McLean C. A. (2010). Characterisation of single nucleotide polymorphisms in the genome of JC polyomavirus using MALDI TOF mass spectrometry. *J. Virol. Methods* 164 63–67. 10.1016/j.jviromet.2009.11.029 19961879

[B17] Benagli C. Rossi V. Dolina M. Tonolla M. Petrini O. (2011). Matrix-assisted laser desorption ionization-time of flight mass spectrometry for the identification of clinically relevant bacteria. *PLoS ONE* 6:e16424 10.1371/journal.pone.0016424 PMC3026826 21283545

[B18] Berrazeg M. Diene S. M. Drissi M. Kempf M. Richet H. Landraud L. (2013). Biotyping of multidrug-resistant Klebsiella pneumoniae clinical isolates from France and Algeria using MALDI-TOF MS. *PLoS ONE* 8:e61428 10.1371/journal.pone.0016428 PMC3631213 23620754

[B19] Bizzini A. Durussel C. Bille J. Greub G. Prod’hom G. (2010). Performance of matrix-assisted laser desorption ionization-time of flight mass spectrometry for identification of bacterial strains routinely isolated in a clinical microbiology laboratory. *J. Clin. Microbiol.* 48 1549–1554. 10.1128/JCM.01794-09 20220166 PMC2863943

[B20] Bizzini A. Jaton K. Romo D. Bille J. Prod’hom G. Greub G. (2011). Matrix-assisted laser desorption ionization–time of flight mass spectrometry as an alternative to 16S rRNA gene sequencing for identification of difficult-To-identify bacterial strains. *J. Clin. Microbiol.* 49 693–696. 10.1128/JCM.01463-10 21106794 PMC3043501

[B21] Böhme K. Fernández-No I. C. Barros-Velázquez J. Gallardo J. M. Calo-Mata P. Cañas B. (2010). Species differentiation of seafood spoilage and pathogenic gram-negative bacteria by MALDI-TOF mass fingerprinting. *J. Proteome Res.* 9 3169–3183. 10.1021/pr100047q 20408567

[B22] Böhme K. Fernández-No I. C. Barros-Velázquez J. Gallardo J. M. Cañas B. Calo-Mata P. (2011). Rapid species identification of seafood spoilage and pathogenic Gram-positive bacteria by MALDI-TOF mass fingerprinting. *Electrophoresis* 32 2951–2965. 10.1002/elps.201100217 22009363

[B23] Böhme K. Fernández-No I. C. Barros-Velázquez J. Gallardo J. M. Cañas B. Calo-Mata P. (2012). SpectraBank: an open access tool for rapid microbial identification by MALDI-TOF MS fingerprinting. *Electrophoresis* 33 2138–2142. 10.1002/elps.201200074 22821489

[B24] Burillo A. Rodríguez-Sánchez B. Ramiro A. Cercenado E. Rodríguez-Créixems M. Bouza E. (2014). Gram-stain plus MALDI-TOF MS (Matrix-Assisted Laser Desorption Ionization-Time of Flight Mass Spectrometry) for a rapid diagnosis of urinary tract infection. *PLoS ONE* 9:e86915 10.1371/journal.pone.0086915 PMC3899310 24466289

[B25] Calderaro A. Arcangeletti M. C. Rodighiero I. Buttrini M. Gorrini C. Motta F. (2014). Matrix-assisted laser desorption/ionization time-of-flight (MALDI-TOF) mass spectrometry applied to virus identification. *Sci. Rep.* 4 6803 10.1038/srep06803 PMC4213803 25354905

[B26] Carbonnelle E. Beretti J. L. Cottyn S. Quesne G. Berche P. Nassif X. (2007). Rapid identification of *Staphylococci* isolated in clinical microbiology laboratories by matrix-assisted laser desorption ionization-time of flight mass spectrometry. *J. Clin. Microbiol.* 45 2156–2161. 10.1128/JCM.02405-06 17507519 PMC1932985

[B27] Carbonnelle E. Grohs P. Jacquier H. Day N. Tenza S. Dewailly A. (2012). Robustness of two MALDI-TOF mass spectrometry systems for bacterial identification. *J. Microbiol. Methods* 89 133–136. 10.1016/j.mimet.2012.03.003 22425492

[B28] Cash P. (2009). Proteomics in the study of the molecular taxonomy and epidemiology of bacterial pathogens. *Electrophoresis* 1 S133–S141. 10.1002/elps.200900059 19517493

[B29] Cassagne C. Pratlong F. Jeddi F. Benikhlef R. Aoun K. Normand A. C. (2014). Identification of *Leishmania* at the species level with matrix-assisted laser desorption ionization time-of-flight mass spectrometry. *Clin. Microbiol. Infect.* 20 551–557. 10.1111/1469-0691.12387 24165542

[B30] Chen H. Y. Chen Y. C. (2005). Characterization of intact *Penicillium* spores by matrix-assisted laser desorption/ionization mass spectrometry. *Rapid Commun. Mass Spectrom.* 19 3564–3568. 10.1002/rcm.2229 16276495

[B31] Cherkaoui A. Emonet S. Fernandez J. Schorderet D. Schrenzel J. (2011). Evaluation of matrix-assisted laser desorption ionization-time of flight mass spectrometry for rapid identification of Beta-hemolytic streptococci. *J. Clin. Microbiol.* 49 3004–3005. 10.1128/JCM.00240-11 21697322 PMC3147758

[B32] Cherkaoui A. Hibbs J. Emonet S. Tangomo M. Girard M. Francois P. (2010). Comparison of two matrix-assisted laser desorption ionization-time of flight mass spectrometry methods with conventional phenotypic identification for routine identification of bacteria to the species level. *J. Clin. Microbiol.* 48 1169–1175. 10.1128/JCM.01881-09 20164271 PMC2849558

[B33] Chou T. C. Hsu W. Wang C. H. Chen Y. J. Fang J. M. (2011). Rapid and specific influenza virus detection by functionalized magnetic nanoparticles and mass spectrometry. *J. Nanobiotechnol.* 9 52 10.1186/1477-3155-9-52 PMC3248366 22088100

[B34] Christner M. Rohde H. Wolters M. Sobottka I. Wegscheider K. Aepfelbacher M. (2010). Rapid identification of bacteria from positive blood culture bottles by use of matrix-assisted laser desorption-ionization time of flight mass spectrometry fingerprinting. *J. Clin. Microbiol.* 48 1584–1591. 10.1128/JCM.01831-09 20237093 PMC2863888

[B35] Couderc C. Nappez C. Drancourt M. (2012). Comparing inactivation protocols of *Yersinia organisms* for identification with matrix-assisted laser desorption/ionization time-of-flight mass spectrometry. *Rapid Commun. Mass Spectrom.* 26 710–714. 10.1002/rcm.6152 22328226

[B36] Croxatto A. Prod’hom G. Greub G. (2012). Applications of MALDI-TOF mass spectrometry in clinical diagnostic microbiology. *FEMS Microbiol. Rev.* 36 380–407. 10.1111/j.1574-6976.2011.00298.x 22092265

[B37] Demarco M. L. Burnham C. A. (2014). Diafiltration MALDI-TOF mass spectrometry method for culture-independent detection and identification of pathogens directly from urine specimens. *Am. J. Clin. Pathol.* 141 204–212. 10.1309/AJCPQYW3B6JLKILC 24436267

[B38] Dieckmann R. Graeber I. Kaesler I. Szewzyk U. von Döhren H. (2005). Rapid screening and dereplication of bacterial isolates from marine sponges of the sula ridge by intact-cell-MALDI-TOF mass spectrometry (ICM-MS). *Appl. Microbiol. Biotechnol.* 67 539–548. 10.1007/s00253-004-1812-2 15614563

[B39] Dieckmann R. Malorny B. (2011). Rapid screening of epidemiologically important *Salmonella enterica* subsp. enterica serovars by whole-cell matrix-assisted laser desorption ionization-time of flight mass spectrometry. *Appl. Environ. Microbiol.* 77 4136–4146. 10.1128/AEM.02418-10 21515723 PMC3131644

[B40] Dong H. Kemptner J. Marchetti-Deschmann M. Kubicek C. P. Allmaier G. (2009). Development of a MALDI two-layer volume sample preparation technique for analysis of colored conidia spores of *Fusarium* by MALDI linear TOF mass spectrometry. *Anal. Bioanal. Chem.* 395 1373–1383. 10.1007/s00216-009-3067-3 19727682

[B41] Donohue M. J. Best J. M. Smallwood A. W. Kostich M. Rodgers M. Shoemaker J. A. (2007). Differentiation of *Aeromonas* isolated from drinking water distribution systems using matrix-assisted laser desorption/ionization-mass spectrometry. *Anal. Chem.* 79 1939–1946. 10.1021/ac0611420 17269751

[B42] Donohue M. J. Smallwood A. W. Pfaller S. Rodgers M. Shoemaker J. A. (2006). The development of a matrix-assisted laser desorption/ionization mass spectrometry-based method for the protein fingerprinting and identification of *Aeromonas* species using whole cells. *J. Microbiol. Methods* 65 380–389. 10.1016/j.mimet.2005.08.005 16176841

[B43] Downard K. M. (2013). Proteotyping for the rapid identification of influenza virus and other biopathogens. *Chem. Soc. Rev.* 42 8584–8595. 10.1039/c3cs60081e 23632861

[B44] Dubois D. Leyssene D. Chacornac J. P. Kostrzewa M. Schmit P. O. Talon R. (2010). Identification of a variety of *Staphylococcus* species by matrix-assisted laser desorption ionization-time of flight mass spectrometry. *J. Clin. Microbiol.* 48 941–945. 10.1128/JCM.00413-09 20032251 PMC2832446

[B45] EI Khéchine A. Couderc C. Flaudrops C. Raoult D. Drancourt M. (2011). Matrix-assisted laser desorption/ionization time-of-flight mass spectrometry identification of mycobacteria in routine clinical practice. *PLoS ONE* 6:e24720 10.1371/journal.pone.0024720 PMC3172293 21935444

[B46] Eddabra R. Prévost G. Scheftel J. M. (2012). Rapid discrimination of environmental *Vibrio* by matrix-assisted laser desorption ionization time-of-flight mass spectrometry. *Microbiol. Res.* 167 226–230. 10.1016/j.micres.2011.09.002 22015259

[B47] Ekström S. Onnerfjord P. Nilsson J. Bengtsson M. Laurell T. Marko-Varga G. (2000). Integrated microanalytical technology enabling rapid and automated protein identification. *Anal. Chem.* 72 286–293. 10.1021/ac990731l 10658321

[B48] Emonet S. Shah H. N. Cherkaoui A. Schrenzel J. (2010). Application and use of various mass spectrometry methods in clinical microbiology. *Clin. Microbiol. Infect.* 16 1604–1613. 10.1111/j.1469-0691.2010.03368.x 20969670

[B49] Erhard M. Hipler U. C. Burmester A. Brakhage A. A. Wöstemeyer J. (2008). Identification of dermatophyte species causing onychomycosis and tinea pedis by MALDI-TOF mass spectrometry. *Exp. Dermatol.* 17 356–361. 10.1111/j.1600-0625.2007.00649.x 17979969

[B50] Espinal P. Seifert H. Dijkshoorn L. Vila J. Roca I. (2011). Rapid and accurate identification of genomic species from the *Acinetobacter baumannii* (Ab) group by MALDI-TOF MS. *Clin. Microbiol. Infect.* 18 1097–1103. 10.1111/j.1469-0691.2011.03696.x 22085042

[B51] Everley R. A. Mott T. M. Wyatt S. A. Toney D. M. Croley T. R. (2008). Liquid chromatography/mass spectrometry characterization of *Escherichia coli* and *Shigella* species. *J. Am. Soc. Mass Spectrom.* 19 1621–1628. 10.1016/j.jasms.2008.07.003 18692404

[B52] Fagerquist C. K. Garbus B. R. Miller W. G. Williams K. E. Yee E. Bates A. H. (2010). Rapid identification of protein biomarkers of *Escherichia coli* O157:H7 by matrix-assisted laser desorption ionization-time-of-flight-time-of-flight mass spectrometry and top-down proteomics. *Anal. Chem.* 82 2717–2725. 10.1021/ac902455d 20232878

[B53] Feli G. Dellaglio F. (2007). On the species descriptions based on a single strain: proposal to introduce the status species proponenda (sp. pr.). *Int. J. Syst. Evol. Microbiol.* 57 2185–2187. 10.1099/ijs.0.64931-0 17766896

[B54] Feltens R. Görner R. Kalkhof S. Gröger-Arndt H. von Bergen M. (2010). Discrimination of different species from the genus *Drosophila* by intact protein profiling using matrix-assisted laser desorption ionization mass spectrometry. *BMC Evol. Biol.* 10:95 10.1186/1471-2148-10-95 PMC2858148 20374617

[B55] Fenselau C. Demirev P. A. (2001). Characterization of intact microorganisms by MALDI mass spectrometry. *Mass. Spectrom. Rev.* 20 157–171. 10.1002/mas.10004 11835304

[B56] Fernandes N. D. Downard K. M. (2014). Origins of the reassortant 2009 pandemic influenza virus through proteotyping with mass spectrometry. *J. Mass Spectrom.* 49 93–102. 10.1002/jms.3310 24446268

[B57] Fernández-No I. C. Böhme K. Gallardo J. M. Barros-Velázquez J. Cañas B. Calo-Mata P. (2010). Differential characterization of biogenic amine-producing bacteria involved in food poisoning using MALDI-TOF mass fingerprinting. *Electrophoresis* 31 1116–1127. 20151397 10.1002/elps.200900591

[B58] Ferreira L. Sánchez-Juanes F. García-Fraile P. Rivas R. Mateos P. F. Martínez-Molina E. (2011). MALDI-TOF mass spectrometry is a fast and reliable platform for identification and ecological studies of species from family Rhizobiaceae. *PLoS ONE* 6:e20223 10.1371/journal.pone.0020223 PMC3105015 21655291

[B59] Ferreira L. Sánchez-Juanes F. González-Avila M. Cembrero-Fuciños D. Herrero-Hernández A. González-Buitrago J. M. (2010). Direct identification of urinary tract pathogens from urine samples by matrix-assisted laser desorption ionization-time of flight mass spectrometry. *J. Clin. Microbiol.* 48 2110–2115. 10.1128/JCM.02215-09 20392910 PMC2884468

[B60] Foster A. G. (2013). Rapid Identification of microbes in positive blood cultures by use of the vitek MS matrix-assisted laser desorption ionization-time of flight mass spectrometry system. *J. Clin. Microbiol.* 51 3717–3719. 10.1128/JCM.01679-13 23985920 PMC3889756

[B61] Friedrichs C. Rodloff A. C. Chhatwal G. S. Schellenberger W. Eschrich K. (2007). Rapid identification of *Viridans Streptococci* by mass spectrometric discrimination. *J. Clin. Microbiol.* 45 2392–2397. 10.1128/JCM.00556-07 17553974 PMC1951256

[B62] Ganova-Raeva L. Ramachandran S. Honisch C. Forbi J. C. Zhai X. Khudyakov Y. (2010). Robust hepatitis B virus genotyping by mass spectrometry. *J. Clin. Microbiol.* 48 4161–4168. 10.1128/JCM.00813-10 20810764 PMC3020871

[B63] Gibb S. Strimmer K. (2012). MALDIquant: a versatile R package for the analysis of mass spectrometry data. *Bioinformatics* 28 2270–2271. 10.1093/bioinformatics/bts447 22796955

[B64] Griffin P. M. Price G. R. Schooneveldt J. M. Schlebusch S. Tilse M. H. Urbanski T. (2012). Use of matrix-assisted laser desorption ionization-time of flight mass spectrometry to identify vancomycin-resistant enterococci and investigate the epidemiology of an outbreak. *J. Clin. Microbiol.* 50 2918–2931. 10.1128/JCM.01000-12 22740710 PMC3421795

[B65] Guembe M. Rodríguez-Sánchez B. Ruiz A. Martín-Rabadán P. Rodríguez-Créixems M. Bouza E. (2014). Can MALDI-TOF mass spectrometry be used with intravascular catheters? *Enferm. Infecc. Microbiol. Clin.* 32 372–374. 10.1016/j.eimc.2014.01.011 24679820

[B66] Haag A. Taylor M. S. N. Johnston K. H. Cole R. B. (1998). Rapid identification and speciation of aemophilus bacteria by matrix-assisted laser desorption/ionization time-of-flight mass spectrometry. *J. Mass Spectrom.* 33 750–756. 10.1002/(SICI)1096-9888(199808)33:8<750::AID-JMS680>3.0.CO;2-1 9745723

[B67] Haigh J. D. Green I. M. Ball D. Eydmann M. Millar M. Wilks M. (2013). Rapid identification of bacteria from bioMérieux BacT/ALERT blood culture bottles by MALDI-TOF MS. *Br. J. Biomed. Sci.* 70 149–155. 24400426 10.1080/09674845.2013.11669949

[B68] Hart P. J. Wey E. McHugh T. D. Balakrishnan I. Belgacem O. (2015). A method for the detection of antibiotic resistance markers in clinical strains of *Escherichia coli* using MALDI mass spectrometry. *J. Microbiol. Methods* 111 1–8. 10.1016/j.mimet.2015.01.020 25633625

[B69] Hazen T. H. Martinez R. J. Chen Y. Lafon P. C. Garrett N. M. Parsons M. B. (2009). Rapid identification of *Vibrio parahaemolyticus* by whole-cell matrix-assisted laser desorption ionization-time of flight mass spectrometry. *Appl. Environ. Microbiol.* 75 6745–6756. 10.1128/AEM.01171-09 19749061 PMC2772414

[B70] He Y. Chang T. C. Li H. Shi G. Tang Y. W. (2011). Matrix-assisted laser desorption ionization time-of-flight mass spectrometry and database for identification of *Legionella* species. *Can. J. Microbiol.* 57 533–538. 10.1139/w11-039 21767077

[B71] He Y. Li H. Lu X. Stratton C. W. Tang Y. W. (2010). Mass spectrometry biotyper system identifies enteric bacterial pathogens directly from colonies grown on selective stool culture media. *J. Clin. Microbiol.* 48 3888–3892. 10.1128/JCM.01290-10 20844226 PMC3020868

[B72] Hettick J. M. Green B. J. Buskirk A. D. Kashon M. L. Slaven J. E. Janotka E. (2008a). Discrimination of *Penicillium* isolates by matrix-assisted laser desorption/ionization time of- flight mass spectrometry fingerprinting. *Rapid Commun. Mass Spectrom.* 22 2555–2560. 10.1002/rcm.3649 18646251

[B73] Hettick J. M. Green B. J. Buskirk A. D. Kashon M. L. Slaven J. E. Janotka E. (2008b). Discrimination of *Aspergillus* isolates at the species and strain level by matrix-assisted laser desorption/ionization time-of-flight mass spectrometry fingerprinting. *Anal. Biochem.* 380 276–281. 10.1016/j.ab.2008.05.051 18577370

[B74] Hettick J. M. Kashon M. L. Simpson J. P. Siegel P. D. Mazurek G. H. Weissman D. N. (2004). Proteomic profiling of intact mycobacteria by matrix-assisted laser desorption/ionization time-of-flight mass spectrometry. *Anal. Chem.* 76 5769–5776. 10.1021/ac049410m 15456297

[B75] Hijazin M. Alber J. Lämmler C. Weitzel T. Hassan A. A. Timke M. (2011). Identification of Trueperella (Arcanobacterium) bernardiae by matrix-assisted laser desorption/ionization time-of-flight mass spectrometry analysis and by species-specific PCR. *J. Med. Microbiol.* 61 457–459. 10.1099/jmm.0.035774-0 22096130

[B76] Hooff G. P. van Kampen J. J. Meesters R. J. van Belkum A. Goessens W. H. Luider T. M. (2012). Characterization of β-lactamase enzyme activity in bacterial lysates using MALDI-mass spectrometry. *J. Proteome Res.* 11 79–84. 10.1021/pr200858r 22013912

[B77] Hoppenheit A. Murugaiyan J. Bauer B. Steuber S. Clausen P.-H. Roesler U. (2013). Identification of Tsetse (*Glossina spp.*) using matrix-assisted laser desorption/ionisation time of flight mass spectrometry. *PLoS Negl. Trop. Dis.* 7:e2305 10.1371/journal.pntd.0002305 PMC3708848 23875040

[B78] Horneffer V. Forsmann A. Strupat K. Hillenkamp F. Kubitscheck U. (2001). Localization of analyte molecules in MALDI preparations by confocal laser scanning microscopy. *Anal. Chem.* 73 1016–1022. 10.1021/ac000499f 11289411

[B79] Hoyos-Mallecot Y. Cabrera-Alvargonzalez J. J. Miranda-Casas C. Rojo-Martín M. D. Liebana-Martos C. Navarro-Marí J. M. (2014). MALDI-TOF MS, a useful instrument for differentiating metallo-β-lactamases in *Enterobacteriaceae* and *Pseudomonas* spp. *Lett. Appl. Microbiol.* 58 325–329. 10.1111/lam.12203 24286119

[B80] Hrabák J. Walková R. Studentová V. Chudácková E. Bergerová T. (2011). Carbapenemase activity detection by matrix-assisted laser desorption ionization-time of flight mass spectrometry. *J. Clin. Microbiol.* 49 3222–3227. 10.1128/JCM.00984-11 21775535 PMC3165603

[B81] Idelevich E. A. Schüle I. Grünastel B. Wüllenweber J. Peters G. Becker K. (2014). Rapid identification of microorganisms from positive blood cultures by MALDI-TOF mass spectrometry subsequent to very short-term incubation on solid medium. *Clin. Microbiol. Infect.* 20 1001–1006. 10.1111/1469-0691.12640 24698361

[B82] Ilina E. N. Borovskaya A. D. Malakhova M. M. Vereshchagin V. A. Kubanova A. A. Kruglov A. N. (2009). Direct bacterial profiling by matrix-assisted laser desorption-ionization time-of-flight mass spectrometry for identification of pathogenic *Neisseria*. *J. Mol. Diagn.* 11 75–86. 10.2353/jmoldx.2009.080079 19095774 PMC2607569

[B83] Ilina E. N. Borovskaya A. D. Serebryakova M. V. Chelysheva V. V. Momynaliev K. T. Maier T. (2010). Application of matrix-assisted laser desorption/ionization time-of-flight mass spectrometry for the study of *Helicobacter pylori*. *Rapid Commun. Mass Spectrom.* 24 328–334. 10.1002/rcm.4394 20049887

[B84] Ilina E. N. Malakhova M. V. Generozov E. V. Nikolaev E. N. Govorun V. M. (2005). Matrix-assisted laser desorption ionization-time of flight (mass spectrometry) for hepatitis C virus genotyping. *J. Clin. Microbiol.* 43 2810–2815. 10.1128/JCM.43.6.2810-2815.2005 15956402 PMC1151883

[B85] Jeong Y. S. Choi S. Chong E. Kim J. H. Kim S. J. (2014). Rapid detection of *Bacillus* spore aerosol particles by direct in situ analysis using MALDI-TOF mass spectrometry. *Lett. Appl. Microbiol.* 59 177–178. 10.1111/lam.12261 24702137

[B86] Jeong Y. S. Lee J. Kim S. J. (2013). Discrimination of *Bacillus anthracis* spores by direct in-situ analysis of matrix-assisted laser desorption/ionization time-of-flight mass spectrometry. *Bull Korean Chem. Soc.* 34 2635–2639. 10.5012/bkcs.2013.34.9.2635

[B87] Johansson A. Nagy E. Sóki J. ESGAI (ESCMID Study Group on Anaerobic Infections). (2014). Detection of carbapenemase activities of *Bacteroides fragilis* strains with matrix-assisted laser desorption ionization–time of flight mass spectrometry (MALDI-TOF MS). *Anaerobe* 26 49–52. 10.1016/j.anaerobe.2014.01.006 24480431

[B88] Jungblut P. R. Hecker M. (2004). Proteomics of microbial pathogens. *Proteomics* 4 2829–2830. 10.1002/pmic.200490063

[B89] Kemptner J. Marchetti-Deschmann M. Mach R. Druzhinina I. S. Kubicek C. P. Allmaier G. (2009). Evaluation of matrix-assisted laser desorption/ionization (MALDI) preparation techniques for surface characterization of intact *Fusarium* spores by MALDI linear time-of-flight mass spectrometry. *Rapid Commun. Mass Spectrom.* 23 877–884. 10.1002/rcm.3949 19224532

[B90] Kiehntopf M. Melcher F. Hänel I. Eladawy H. Tomaso H. (2011). Differentiation of *Campylobacter* species by surface-enhanced laser desorption/ionization-time-of-flight mass spectrometry. *Foodborne Pathog. Dis.* 8 875–885. 10.1089/fpd.2010.0775 21524195

[B91] Kilian M. Poulsen K. Blomqvist T. Håvarstein L. S. Bek-Thomsen M. Tettelin H. (2008). Evolution of *Streptococcus pneumoniae* and its close commensal relatives. *PLoS ONE* 3:e2683 10.1371/journal.pone.0002683 PMC2444020 18628950

[B92] Kliem M. Sauer S. (2012). The essence on mass spectrometry based microbial diagnostics. *Curr. Opin. Microbiol.* 15 397–402. 10.1016/j.mib.2012.02.006 22410108

[B93] Köhling H. L. Bittner A. Müller K. D. Buer J. Becker M. Rübben H. (2012). Direct identification of bacteria in urine samples by matrix-assisted laser desorption/ionization time-of-flight mass spectrometry and relevance of defensins as interfering factors. *J. Med. Microbiol.* 61 339–344. 10.1099/jmm.0.032284-0 22275503

[B94] Kostrzewa M. Sparbier K. Maier T. Schubert S. (2013). MALDI-TOF MS: an upcoming tool for rapid detection of antibiotic resistance in microorganisms. *Proteomics Clin. Appl.* 7 767–778. 10.1002/prca.201300042 24123965

[B95] Kuhnert P. Bisgaard M. Korczak B. M. Schwendener S. Christensen H. Frey J. (2012). Identification of animal Pasteurellaceae by MALDI-TOF mass spectrometry. *J. Microbiol. Methods* 89 1–7. 10.1016/j.mimet.2012.02.001 22343217

[B96] Kull S. Pauly D. Störmann B. Kirchner S. Stämmler M. Dorner M. B. (2010). Multiplex detection of microbial and plant toxins by immunoaffinity enrichment and matrix-assisted laser desorption/ionization mass spectrometry. *Anal. Chem.* 82 2916–2924. 10.1021/ac902909r 20199054

[B97] Kumar M. P. Vairamani M. Raju R. P. Lobo C. Anbumani N. Kumar C. P. (2004). Rapid discrimination between strains of beta haemolytic streptococci by intact cell mass spectrometry. *Indian J. Med. Res.* 119 283–288. 15243166

[B98] La Scola B. Raoult D. (2009). Direct identification of bacteria in positive blood culture bottles by matrix-assisted laser desorption ionisation time-of-flight mass spectrometry. *PLoS ONE* 4:e8041 10.1371/journal.pone.0008041 PMC2777307 19946369

[B99] Lamy B. Kodjo A. Laurent F. ColBVH Study Group. (2011). Identification of *Aeromonas* isolates by matrix-assisted laser desorption ionization time-of-flight mass spectrometry. *Diagn. Microbiol. Infect. Dis.* 71 1–5. 10.1016/j.diagmicrobio.2011.04.014 21763094

[B100] Lartigue M. F. Héry-Arnaud G. Haguenoer E. Domelier A. S. Schmit P. O. van der Mee-Marquet N. (2009). Identification of *Streptococcus agalactiae* isolates from various phylogenetic lineages by matrix-assisted laser desorption ionization-time of flight mass spectrometry. *J. Clin. Microbiol.* 47 2284–2287. 10.1128/JCM.00175-09 19403759 PMC2708490

[B101] Lasch P. Beyer W. Nattermann H. Stämmler M. Siegbrecht E. Grunow R. (2009). Identification of *Bacillus anthracis* by using matrix-assisted laser desorption ionization-time of flight mass spectrometry and artificial neural networks. *Appl. Environ. Microbiol.* 75 7229–7242. 10.1128/AEM.00857-09 19767470 PMC2786504

[B102] Lasch P. Nattermann H. Erhard M. Stämmler M. Grunow R. Bannert N. (2008). MALDI-TOF mass spectrometry compatible inactivation method for highly pathogenic microbial cells and spores. *Anal. Chem.* 80 2026–2034. 10.1021/ac701822j 18290666

[B103] Lau A. F. Drake S. K. Calhoun L. B. Henderson C. M. Zelazny A. M. (2013). Development of a clinically comprehensive database and a simple procedure for identification of molds from solid media by matrix-assisted laser desorption ionization–time of flight mass spectrometry. *J. Clin. Microbiol.* 51 828–834. 10.1128/JCM.02852-12 23269728 PMC3592033

[B104] Lavergne R. A. Chauvin P. Valentin A. Fillaux J. Roques-Malecaze C. Arbnaud S. (2013). An extraction method of positive blood cultures for direct identification of *Candida species* by Vitek MS matrix-assisted laser desorption ionization time of flight mass spectrometry. *Med. Mycol.* 51 652–656. 10.3109/13693786.2012.762607 23373445

[B105] Lay J. O. Jr. (2001). MALDI-TOF mass spectrometry of bacteria. *Mass Spectrom. Rev.* 20 172–194. 10.1002/mas.10003 11835305

[B106] Lefmann M. Honisch C. Böcker S. Storm N. von Wintzingerode F. Schlötelburg C. (2004). Novel mass spectrometry-based tool for genotypic identification of mycobacteria. *J. Clin. Microbiol.* 42 339–346. 10.1128/JCM.42.1.339-346.2004 14715774 PMC321663

[B107] Li T. Y. Liu B. H. Chen Y. C. (2000). Characterization of *Aspergillus spores* by matrix-assisted laser desorption/ionization time-of-flight mass spectrometry. *Rapid Commun. Mass Spectrom.* 14 2393–2400. 10.1002/1097-0231(20001230)14:24<2393::AID-RCM178>3.0.CO;2-9 11114056

[B108] Lista F. Reubsaet F. A. De Santis R. Parchen R. R. de Jong A. L. Kieboom J. (2011). Reliable identification at the species level of *Brucella isolates* with MALDI-TOF-MS. *BMC Microbiol.* 11:267 10.1186/1471-2180-11-267 PMC3314589 22192890

[B109] Luan J. Yuan J. Li X. Jin S. Yu L. Liao M. (2009). Multiplex detection of 60 hepatitis B virus variants by maldi-tof mass spectrometry. *Clin. Chem.* 55 1503–1509. 10.1373/clinchem.2009.124859 19541863

[B110] Lundquist M. Caspersen M. B. Wikström P. Forsman M. (2005). Discrimination of *Francisella tularensis* subspecies using surface enhanced laser desorption ionization mass spectrometry and multivariate data analysis. *FEMS Microbiol. Lett.* 243 303–310. 10.1016/j.femsle.2004.12.020 15668033

[B111] Luo Y. Siu G. K. Yeung A. S. Chen J. H. Ho P. L. Leung K. W. (2015). Performance of the VITEK MS matrix-assisted laser desorption ionization-time of flight mass spectrometry system for rapid bacterial identification in two diagnostic centers in China. *J. Med. Microbiol.* 64 18–24. 10.1099/jmm.0.080317-0 25418737

[B112] Mc Taggart L. R. Lei E. Richardson S. E. Hoang L. Fothergill A. Zhang S. X. (2011). Rapid identification of *Cryptococcus neoformans* and *Cryptococcus gattii* by matrix-assisted laser desorption ionization-time of flight mass spectrometry. *J. Clin. Microbiol.* 49 3050–3053. 10.1128/JCM.00651-11 21653762 PMC3147715

[B113] Mellmann A. Cloud J. Maier T. Keckevoet U. Ramminger I. Iwen P. (2008). Evaluation of matrix-assisted laser desorption ionization–time-of-flight mass spectrometry in comparison to 16S rRNA gene sequencing for species identification of nonfermenting bacteria. *J. Clin. Microbiol.* 46 1946–1954. 10.1128/JCM.00157-08 18400920 PMC2446840

[B114] Mencacci A. Monari C. Leli C. Merlini L. De Carolis E. Vella A. (2013). Typing of nosocomial outbreaks of *Acinetobacter baumannii* by use of matrix-assisted laser desorption ionization-time of flight mass spectrometry. *J. Clin. Microbiol.* 51 603–606. 10.1128/JCM.01811-12 23175257 PMC3553918

[B115] Munoz R. López-López A. Urdiain M. Moore E. R. Rosselló-Móra R. (2011). Evaluation of matrix-assisted laser desorption ionization-time of flight whole cell profiles for assessing the cultivable diversity of aerobic and moderately halophilic prokaryotes thriving in solar saltern sediments. *Syst. Appl. Microbiol.* 34 69–75. 10.1016/j.syapm.2010.11.012 21242046

[B116] Muroi M. Shima K. Nakagawa Y. Tanamoto K. (2011). Application of matrix-assisted laser desorption ionization-time of flight mass spectrometry for discrimination of *Escherichia* strains possessing highly conserved ribosomal RNA gene sequences. *Biol. Pharm. Bull.* 34 430–432. 10.1248/bpb.34.430 21372397

[B117] Murray P. R. (2012). What is new in clinical microbiology-microbial identification by MALDI-TOF mass spectrometry. *J. Mol. Diagn.* 14 419–423. 10.1016/j.jmoldx.2012.03.007 22795961 PMC3427873

[B118] Nagy E. Maier T. Urban E. Terhes G. Kostrzewa M. ESCMID Study Group on Antimicrobial Resistance in Anaerobic Bacteria. (2009). Species identification of clinical isolates of *Bacteroides* by matrix-assisted laser-desorption/ionization time-of-flight mass spectrometry. *Clin. Microbiol. Infect.* 15 796–802. 10.1111/j.1469-0691.2009.02788.x 19438622

[B119] Nakano S. Matsumura Y. Kato K. Yunoki T. Hotta G. Noguchi T. (2014). Differentiation of vanA-positive *Enterococcus faecium* from vanA-negative *E. faecium* by matrix-assisted laser desorption/ionisation time-of-flight mass spectrometry. *Int. J. Antimicrob. Agents* 44 256–259. 10.1016/j.ijantimicag.2014.05.006 25104134

[B120] Ndukum J. Atlas M. Datta S. (2011). pkDACLASS: open source software for analyzing MALDI-TOF data. *Bioinformation* 6 45–47. 10.6026/97320630006045 21464846 PMC3064853

[B121] Nenoff P. Erhard M. Simon J. C. Muylowa G. K. Herrmann J. Rataj W. (2013). MALDI-TOF mass spectrometry - a rapid method for the identification of dermatophyte species. *Med. Mycol.* 51 17–24. 10.3109/13693786.2012.685186 22574631

[B122] Nguyen D. T. Van Hoorde K. Cnockaert M. De Brandt E. Aerts M. Binh Thanh L. (2013). A description of the lactic acid bacteria microbiota associated with the production of traditional fermented vegetables in *Vietnam*. *Int. J. Food Microbiol.* 163 19–27. 10.1016/j.ijfoodmicro.2013.01.024 23500611

[B123] Nicolaou N. Xu Y. Goodacre R. (2012). Detection and quantification of bacterial spoilage in milk and pork meat using MALDI-TOF-MS and multivariate analysis. *Anal. Chem.* 84 5951–5958. 10.1021/ac300582d 22698768

[B124] Nilsson C. L. (1999). Fingerprinting of *Helicobacter pylori* strains by matrix assisted laser desorption/ionization mass spectrometric analysis. *Rapid Commun. Mass Spectrom.* 13 1067–1071. 10.1002/(SICI)1097-0231(19990615)13:11<1067::AID-RCM612>3.0.CO;2-N 10368980

[B125] Nørskov-Lauritsen N. Bruun B. Andersen C. Kilian M. (2012). Identification of haemolytic *Haemophilus* species isolated from human clinical specimens and description of *Haemophilus sputorum* sp. nov. *Int. J. Med. Microbiol.* 302 78–83. 10.1016/j.ijmm.2012.01.001 22336150

[B126] O’Leary A. M. Whyte P. Madden R. H. Cormican M. Moore J. E. Mc Namara E. (2011). Pulsed field gel electrophoresis typing of human and retail foodstuff *Campylobacters*: an irish perspective. *Food Microbiol.* 28 426–433. 10.1016/j.fm.2010.10.003 21356447

[B127] Pace N. L. (1997). A molecular view of microbial diversity and the biosphere. *Science* 276 734–740. 10.1126/science.276.5313.734 9115194

[B128] Pan Y. L. Chow N. H. Chang T. C. Chang H. C. (2011). Identification of lethal *Aspergillus* at early growth stages based on matrix-assisted laser desorption/ionization time-of-flight mass spectrometry. *Diagn. Microbiol. Infect. Dis.* 70 344–354. 10.1016/j.diagmicrobio.2011.03.007 21546196

[B129] Patel R. (2015). MALDI-TOF MS for the diagnosis of infectious diseases. *Clin. Chem.* 61 100–111. 10.1373/clinchem.2014.221770 25278500

[B130] Peng J. Yang F. Xiong Z. Guo J. Du J. Hu Y. (2013). Sensitive and rapid detection of viruses associated with hand foot and mouth disease using multiplexed MALDI-TOF analysis. *J. Clin. Virol.* 56 170–174. 10.1016/j.jcv.2012.10.020 23194776

[B131] Perera M. R. Vanstone V. A. Jones M. G. (2005). A novel approach to identify plant parasitic nematodes using matrix-assisted laser desorption/ionization time-of-flight mass spectrometry. *Rapid Commun. Mass Spectrom.* 19 1454–1460. 10.1002/rcm.1943 15880621

[B132] Pfaller M. A. Messer S. A. Hollis R. J. Jones R. N. SENTRY Participants Group. (2002). Antifungal activities of posaconazole, ravuconazole, and voriconazole compared to those of itraconazole and amphotericin B against 239 clinical isolates of *Aspergillus* spp. and other filamentous fungi: report from SENTRY Antimicrobial Surveillance Program, 2000. *Antimicrob. Agents Chemother.* 46 1032–1037. 10.1128/AAC.46.4.1032-1037.2002 11897586 PMC127116

[B133] Piao J. Jiang J. Xu B. Wang X. Guan Y. Wu W. (2012). Simultaneous detection and identification of enteric viruses by PCR-mass assay. *PLoS ONE* 7:e42251 10.1371/journal.pone.0042251 PMC3411642 22870310

[B134] Pierce C. Y. Barr J. R. Woolfitt A. R. Moura H. Shaw E. I. Thompson H. A. (2007). Strain and phase identification of the U.S. category B agent *Coxiella burnetii* by matrix assisted laser desorption/ionization time-of-flight mass spectrometry and multivariate pattern recognition. *Anal. Chim. Acta* 583 23–31. 10.1016/j.aca.2006.09.065 17386522

[B135] Prod’hom G. Bizzini A. Durussel C. Bille J. Greub G. (2010). Matrix-assisted laser desorption ionization-time of flight mass spectrometry for direct bacterial identification from positive blood culture pellets. *J. Clin. Microbiol.* 48 1481–1483. 10.1128/JCM.01780-09 20164269 PMC2849571

[B136] Pulcrano G. Roscetto E. Iula V. D. Panellis D. Rossano F. Catania M. R. (2012). MALDI-TOF mass spectrometry and microsatellite markers to evaluate *Candida parapsilosis* transmission in neonatal intensive care units. *Eur. J. Clin. Microbiol. Infect. Dis.* 31 2919–2928. 10.1007/s10096-012-1642-6 22644055

[B137] Qian J. Cutler J. E. Cole R. B. Cai Y. (2008). MALDI-TOF mass signatures for differentiation of yeast species, strain grouping and monitoring of morphogenesis markers. *Anal. Bioanal. Chem.* 392 439–449. 10.1007/s00216-008-2288-1 18690424

[B138] Quirino A. Pulcrano G. Rametti L. Puccio R. Marascio N. Catania M. R. (2014). Typing of *Ochrobactrum anthropi* clinical isolates using automated repetitive extragenic palindromic-polymerase chain reaction DNA fingerprinting and matrix-assisted laser desorption/ionization-time-of-flight mass spectrometry. *BMC Microbiol.* 14:74 10.1186/1471-2180-14-74 PMC3977698 24655432

[B139] Raus M. Šebela M. (2013). BIOSPEAN: a freeware tool for processing spectra from MALDI intact cell/spore mass spectrometry. *J. Proteomics. Bioinform.* 6 282–287.

[B140] Rosselló G. A. Rodríguez M. P. de Lejarazu Leonardo R. O. Domingo A. O. Pérez M. A. (2014). New procedure for rapid identification of microorganisms causing urinary tract infection from urine samples by mass spectrometry (MALDI-TOF). *Enferm. Infecc. Microbiol. Clin.* 33 89–94. 10.1016/j.eimc.2014.02.022 24796945

[B141] Ruelle V. EI Moualij B. Zorzi W. Ledent P. Pauw E. D. (2004). Rapid identification of environmental bacterial strains by matrix-assisted laser desorption/ionization time-of-flight mass spectrometry. *Rapid Commun. Mass Spectrom.* 18 2013–2019. 10.1002/rcm.1584 15378711

[B142] Saffert R. T. Cunningham S. A. Ihde S. M. Kristine E. Monson J. Patel R. (2011). Comparison of bruker biotyper matrix-assisted laser desorption ionization–time of flight mass spectrometer to BD phoenix automated microbiology system for identification of gram-negative bacilli. *J. Clin. Microbiol.* 49 887–892. 10.1128/JCM.01890-10 21209160 PMC3067728

[B143] Saleeb P. G. Drake S. K. Murray P. R. Zelazny A. M. (2011). Identification of mycobacteria in solid-culture media by matrix-assisted laser desorption ionization-time of flight mass spectrometry. *J. Clin. Microbiol.* 49 1790–1794. 10.1128/JCM.02135-10 21411597 PMC3122647

[B144] Samson R. A. Varga J. (2009). What is a species in *Aspergillus*? *Med. Mycol.* 47 S13–S20. 10.1080/13693780802354011 19255907

[B145] Santos C. Paterson R. R. Venâncio A. Lima N. (2010). Filamentous fungal characterizations by matrix-assisted laser desorption/ionization time-of-flight mass spectrometry. *J. Appl. Microbiol.* 108 375–385. 10.1111/j.1365-2672.2009.04448.x 19659699

[B146] Schröd W. Heydel T. Schwartze V. U. Hoffmann K. Grosse-Herrenthey A. Walther G. (2012). Direct analysis and identification of pathogenic *Lichtheimia* species by matrix-assisted laser desorption ionization-time of flight analyzer-mediated mass spectrometry. *J. Clin. Microbiol.* 50 419–427. 10.1128/JCM.01070-11 22135259 PMC3264171

[B147] Schwahn A. B. Wong J. W. Downard K. M. (2009). Subtyping of the influenza virus by high resolution mass spectrometry. *Anal. Chem.* 81 3500–3506. 10.1021/ac900026f 19402721

[B148] Schwahn A. B. Wong J. W. Downard K. M. (2010). Typing of human and animal strains of influenza virus with conserved signature peptides of matrix M1 protein by high resolution mass spectrometry. *J. Virol. Methods* 165 178–185. 10.1016/j.jviromet.2010.01.015 20117137

[B149] Segawa S. Sawai S. Murata S. Nishimura M. Beppu M. Sogawa K. (2014). Direct application of MALDI-TOF mass spectrometry to cerebrospinal fluid for rapid pathogen identification in a patient with bacterial meningitis. *Clin. Chim. Acta.* 435 59–61. 10.1016/j.cca.2014.04.024 24797349

[B150] Seibold E. Maier T. Kostrzewa M. Zeman E. Splettstoesser W. (2010). Identification of *Francisella tularensis* by whole-cell matrix-assisted laser desorption ionization-time of flight mass spectrometry: fast, reliable, robust, and cost-effective differentiation on species and subspecies levels. *J. Clin. Microbiol.* 48 1061–1069. 10.1128/JCM.01953-09 20181907 PMC2849607

[B151] Sendid B. Ducoroy P. François N. Lucchi G. Spinali S. Vagner O. (2013). Evaluation of MALDI-TOF mass spectrometry for the identification of medically-important yeasts in the clinical laboratories of *Dijon* and *Lille hospitals*. *Med. Mycol.* 51 25–32. 10.3109/13693786.2012.693631 22703164

[B152] Seng P. Drancourt M. Gouriet F. La Scola B. Fournier P. E. Rolain J. M. (2009). Ongoing revolution in bacteriology: routine identification of bacteria by matrix-assisted laser desorption ionization time-of-flight mass spectrometry.C *lin. Infect. Dis.* 49 543–551. 10.1086/600885 19583519

[B153] Seyfarth F. Ziemer M. Sayer H. G. Burmester A. Erhard M. Welker M. (2008). The use of ITS DNA sequence analysis and MALDI-TOF mass spectrometry in diagnosing an infection with *Fusarium proliferatum*. *Exp. Dermatol.* 17 965–971. 10.1111/j.1600-0625.2008.00726.x 18547323

[B154] Shaw E. I. Moura H. Woolfitt A. R. Ospina M. Thompson H. A. Barr J. R. (2004). Identification of biomarkers of whole *Coxiella burnetii* phase I by MALDI-TOF mass spectrometry. *Anal. Chem.* 76 4017–4022. 10.1021/ac030364k 15253637

[B155] Shitikov E. Ilina E. Chernousova L. Borovskaya A. Rukin I. Afanas’ev M. (2012). Mass spectrometry based methods for the discrimination and typing of mycobacteria. *Infect. Genet. Evol.* 12 838–845. 10.1016/j.meegid.2011.12.013 22230718

[B156] Sjöholm M. I. Dillner J. Carlson J. (2008). Multiplex detection of human herpesviruses from archival specimens by using matrix-assisted laser desorption ionization-time of flight mass spectrometry. *J. Clin. Microbiol.* 46 540–545. 10.1128/JCM.01565-07 18094141 PMC2238100

[B157] Spanu T. Posteraro B. Fiori B. D’Inzeo T. Campoli S. Ruggeri A. (2012). Direct maldi-tof mass spectrometry assay of blood culture broths for rapid identification of *Candida* species causing bloodstream infections: an observational study in two large microbiology laboratories. *J. Clin. Microbiol.* 50 176–179. 10.1128/JCM.05742-11 22090401 PMC3256727

[B158] Sparbier K. Schubert S. Weller U. Boogen C. Kostrzewa M. (2012a). Matrix-assisted laser desorption ionization-time of flight mass spectrometry-based functional assay for rapid detection of resistance against β-lactam antibiotics. *J. Clin. Microbiol.* 50 927–937. 10.1128/JCM.05737-11 22205812 PMC3295133

[B159] Sparbier K. Weller U. Boogen C. Kostrzewa M. (2012b). Rapid detection of *Salmonella* sp. by means of a combination of selective enrichment broth and MALDI-TOF MS. *Eur. J. Clin. Microbiol. Infect. Dis.* 31 767–773. 10.1007/s10096-011-1373-0 21842294

[B160] Stackebrandt E. Goebel B. M. (1994). A place for DNA-DNA reassociation and 16S rRNA sequence analysis in the present species definition in bacteriology. *Int. J. Syst. Bacteriol.* 44 846–849. 10.1099/00207713-44-4-846

[B161] Stephan R. Cernela N. Ziegler D. Pflüger V. Tonolla M. Ravasi D. (2011). Rapid species specific identification and subtyping of *Yersinia enterocolitica* by MALDI-TOF mass spectrometry. *J. Microbiol. Methods* 87 150–153. 10.1016/j.mimet.2011.08.016 21914454

[B162] Stephan R. Ziegler D. Pflüger V. Vogel G. Lehner A. (2010). Rapid genus- and species-specific identification of *Cronobacter* spp. by matrix-assisted laser desorption ionization-time of flight mass spectrometry. *J. Clin. Microbiol.* 48 2846–2851. 10.1128/JCM.00156-10 20554814 PMC2916578

[B163] Stevenson L. G. Drake S. K. Murray P. R. (2010a). Rapid identification of bacteria in positive blood culture broths by matrix-assisted laser desorption ionization-time of flight mass spectrometry. *J. Clin. Microbiol.* 48 444–447. 10.1128/JCM.01541-09 19955282 PMC2815598

[B164] Stevenson L. G. Drake S. K. Shea Y. R. Zelazny A. M. Murray P. R. (2010b). Evaluation of matrix-assisted laser desorption ionization-time of flight mass spectrometry for identification of clinically important yeast species. *J. Clin. Microbiol.* 48 3482–3486. 10.1128/JCM.00687-09 20668126 PMC2953094

[B165] Strohalm M. Hassman M. Kosata B. Kodícek M. (2008). mMass data miner: an open source alternative for mass spectrometric data analysis. *Rapid Commun. Mass Spectrom.* 22 905–908. 10.1002/rcm.3444 18293430

[B166] Tadros M. Petrich A. (2013). Evaluation of MALDI-TOF mass spectrometry and Sepsityper Kit^TM^ for the direct identification of organisms from sterile body fluids in a Canadian pediatric hospital. *Can. J. Infect. Dis. Med. Microbiol.* 24 191–194. 24489560 10.1155/2013/701093 PMC3905001

[B167] Theel E. S. Hall L. Mandrekar J. Wengenack N. L. (2011). Dermatophyte identification using matrix-assisted laser desorption ionization-time of flight mass spectrometry. *J. Clin. Microbiol.* 49 4067–4071. 10.1128/JCM.01280-11 21956979 PMC3232958

[B168] Theel E. S. Schmitt B. H. Hall L. Cunningham S. A. Walchak R. C. Patel R. (2012). Formic acid-based direct, on-plate testing of yeast and *Corynebacterium* species by Bruker Biotyper matrix-assisted laserdesorption ionization–time of flight mass spectrometry. *J. Clin. Microbiol.* 50 3093–3095. 10.1128/JCM.01045-12 22760034 PMC3421773

[B169] Tiwari V. Tiwari M. (2014). Quantitative proteomics to study carbapenem resistance in *Acinetobacter baumannii*. *Front. Microbiol.* 5:512 10.3389/fmicb.2014.00512 PMC4176082 25309531

[B170] Torsvik V. L. Øvreas L. Thingstad T. F. (2002). Prokaryotic diversity - magnitude, dynamics, and controlling factors. *Science* 296 1064–1066. 10.1126/science.1071698 12004116

[B171] Uhlik O. Strejcek M. Junkova P. Sanda M. Hroudova M. Vlcek C. (2011). Matrix-assisted laser desorption ionization (MALDI)-time of flight mass spectrometry- and MALDI biotyper-based identification of cultured biphenyl-metabolizing bacteria from contaminated horseradish rhizosphere soil. *Appl. Environ. Microbiol.* 77 6858–6866. 10.1128/AEM.05465-11 21821747 PMC3187117

[B172] Valentine N. Wunschel S. Wunschel D. Petersen C. Wahl K. (2005). Effect of culture conditions on microorganism identification by matrix-assisted laser desorption ionization mass spectrometry. *Appl. Environ. Microbiol.* 71 58–64. 10.1128/AEM.71.1.58-64.2005 15640170 PMC544247

[B173] van Baar B. L. (2000). Characterisation of bacteria by matrix-assisted laser desorption/ionisation and electrospray mass spectrometry. *FEMS Microbiol. Rev.* 24 193–219. 10.1016/S0168-6445(99)00036-4 10717314

[B174] van Veen S. Q. Claas E. C. Kuijper E. J. (2010). High-throughput identification of bacteria and yeast by matrix-assisted laser desorption ionization-time of flight mass spectrometry in conventional medical microbiology laboratories. *J. Clin. Microbiol.* 48 900–907. 10.1128/JCM.02071-09 20053859 PMC2832429

[B175] Vandamme P. Vancanneyt M. van Belkum A. Segers P. Quint W. G. Kersters K. (1996). Polyphasic analysis of strains of the genus *Capnocytophaga* and Centers for Disease Control group DF-3. *Int. J. Syst. Bacteriol.* 46 782–791. 10.1099/00207713-46-3-782 8782690

[B176] Verroken A. Janssens M. Berhin C. Bogaerts P. Huang T. D. Wauters G. (2010). Evaluation of matrix-assisted laser desorption ionization–time of flight mass spectrometry for identification of *Nocardia* species. *J. Clin. Microbiol.* 48 4015–4021. 10.1128/JCM.01234-10 20861335 PMC3020850

[B177] Vila J. Juiz P. Salas C. Almela M. de la Fuente C. G. Zboromyrska Y. (2012). Identification of clinically relevant *Corynebacterium* spp., *Arcanobacterium haemolyticum and Rhodococcus equi* by matrix-assisted laser desorption ionization-time of flight mass spectrometry. *J. Clin. Microbiol.* 50 1745–1747. 10.1128/JCM.05821-11 22337985 PMC3347156

[B178] von Bergen M. Eidner A. Schmidt F. Murugaiyan J. Wirth H. Binder H. (2009). Identification of harmless and pathogenic algae of the genus Prototheca by MALDI-MS. *Proteomics Clin. Appl.* 3 774–784. 10.1002/prca.200780138 21136986

[B179] Vranakis I. Papadioti A. Tselentis Y. Psaroulaki A. Tsiotis G. (2013). The contribution of proteomics towards deciphering the enigma of *Coxiella burnetii*. *Proteomics Clin. Appl.* 7 193–204. 10.1002/prca.201200096 23255407

[B180] Walker J. Fox A. J. Edwards-Jones V. Gordon D. B. (2002). Intact cell mass spectrometry (ICMS) used to type methicillin-resistant *Staphylococcus aureus*: media effects and inter-laboratory reproducibility. *J. Microbiol. Methods* 48 117–126. 10.1016/S0167-7012(01)00316-5 11777562

[B181] Wallet F. LoÃ¯ez C. Decoene C. Courcol R. (2011). Rapid identification of *Cardiobacterium hominis* by MALDI-TOF mass spectrometry during infective endocarditis. *Jpn. J. Infect. Dis.* 64 327–329. 21788710

[B182] Wang J. Chen W. F. Li Q. X. (2012). Rapid identification and classification of *Mycobacterium* spp. using whole-cell protein barcodes with matrix assisted laser desorption ionization time of flight mass spectrometry in comparison with multigene phylogenetic analysis. *Anal. Chim. Acta* 716 133–137. 10.1016/j.aca.2011.12.016 22284888

[B183] Wang L. J. Lu X. X. Wu W. Sui W. J. Zhang G. (2014). Application of matrix-assisted laser desorption ionization time-of-flight mass spectrometry in the screening of vanA-positive *Enterococcus faecium*. *Eur. J. Mass Spectrom.* 20 461–465. 10.1255/ejms.1298 25905870

[B184] Wang Y. Cui S. Li F. (2008). Study on detection and identification of *Salmonella* species by matrix-assisted laser desorption/ionization time-of-flight mass spectrometry. *Wei. Sheng. Yan. Jiu.* 37 685–689. 19239002

[B185] Wang Z. Russon L. Li L. Roser D. C. Long S. R. (1998). Investigation of spectral reproducibility in direct analysis of bacteria proteins by matrix-assisted laser desorption/ionization time-of-flight mass spectrometry. *Rapid Commun. Mass Spectrom.* 12 456–464. 10.1002/(SICI)1097-0231(19980430)12:8<456::AID-RCM177>3.0.CO;2-U 9586233

[B186] Wasinger V. C. Cordwell S. J. Cerpa-Poljak A. Yan J. X. Gooley A. A. Wilkins M. R. (1995). Progress with gene-product mapping of the Mollicutes: *Mycoplasma genitalium*. *Electrophoresis* 16 1090–1094. 10.1002/elps.11501601185 7498152

[B187] Welker M. (2011). Proteomics for routine identification of microorganisms. *Proteomics* 11 3143–3153. 10.1002/pmic.201100049 21726051

[B188] Woese C. Kandler O. Wheelis M. L. (1990). Towards a natural system of organisms: proposals for the domains Achaea, Bacteria, and Eucarya. *Proc. Natl. Acad. Sci. U.S.A.* 87 4576–4579. 10.1073/pnas.87.12.4576 2112744 PMC54159

[B189] Wolters M. Rohde H. Maier T. Belmar-Campos C. Franke G. Scherpe S. (2011). MALDI-TOF MS fingerprinting allows for discrimination of major methicillin-resistant *Staphylococcus aureus* lineages. *Int. J. Med. Microbiol.* 301 64–68. 10.1016/j.ijmm.2010.06.002 20728405

[B190] Wunschel D. S. Hill E. A. McLean J. S. Jarman K. Gorby Y. A. Valentine N. (2005). Effects of varied pH, growth rate and temperature using controlled fermentation and batch culture on matrix assisted laser desorption/ionization whole cell protein fingerprints. *J. Microbiol. Methods* 62 259–271. 10.1016/j.mimet.2005.04.033 15979749

[B191] Yaman G. Akyar I. Can S. (2012). Evaluation of the MALDI TOF-MS method for identification of *Candida* strains isolated from blood cultures. *Diagn. Microbiol. Infect. Dis.* 73 65–67. 10.1016/j.diagmicrobio.2012.01.013 22578939

[B192] Yates J. R. III (1998). Mass spectrometry and the age of the proteome. *J. Mass Spectrom.* 33 1–19. 10.1002/(SICI)1096-9888(199801)33:1<1::AID-JMS624>3.0.CO;2-9 9449829

[B193] Yi X. Li J. Yu S. Zhang A. Xu J. Yi J. (2011). A new PCR-based mass spectrometry system for high-risk HPV, part I: methods. *Am. J. Clin. Pathol.* 136 913–919. 10.1309/AJCPWTZDT0Q7DOVI 22095377

[B194] Yssouf A. Parola P. Lindström A. Lilja T. L’Ambert G. Bondesson U. (2014). Identification of European mosquito species by MALDI-TOF MS. *Parasitol. Res.* 113 2375–2378. 10.1007/s00436-014-3876-y 24737398

[B195] Zaluga J. Heylen K. Van Hoorde K. Hoste B. Van Vaerenbergh J. Maes M. (2011). *GyrB* sequence analysis and MALDI-TOF MS as identification tools for plant pathogenic *Clavibacter*. *Syst. Appl. Microbiol.* 34 400–407. 10.1016/j.syapm.2011.05.001 21802235

[B196] Zhu S. Ratering S. Schnell S. Wacker R. (2011). Matrix-assisted laser desorption and ionization-time-of-flight mass spectrometry, 16S rRNA gene sequencing, and API 32E for identification of *Cronobacter* spp.: a comparative study. *J. Food Prot.* 74 2182–2187. 10.4315/0362-028X.JFP-11-205 22186062

[B197] Zürcher S. Mooser C. Lüthi A. U. Mühlemann K. Barbani M. T. Mohacsi P. (2012). Sensitive and rapid detection of ganciclovir resistance by PCR based MALDI-TOF analysis. *J. Clin. Virol.* 54 359–363. 10.1016/j.jcv.2012.04.019 22633201

